# Not all mosquitoes are created equal: A synthesis of vector competence experiments reinforces virus associations of Australian mosquitoes

**DOI:** 10.1371/journal.pntd.0010768

**Published:** 2022-10-04

**Authors:** Morgan P. Kain, Eloise B. Skinner, Tejas S. Athni, Ana L. Ramirez, Erin A. Mordecai, Andrew F. van den Hurk

**Affiliations:** 1 Department of Biology, Stanford University, Stanford, California, United States of America; 2 Natural Capital Project, Woods Institute for the Environment, Stanford University, Stanford, California, United States of America; 3 Centre for Planetary Health and Food Security, Griffith University, Gold Coast, Queensland, Australia; 4 Department of Pathology, Microbiology, and Immunology, University of California - Davis, Davis, California, United States of America; 5 Public Health Virology, Forensic and Scientific Services, Department of Health, Queensland Government, Brisbane, Queensland, Australia; Connecticut Agricultural Experiment Station, UNITED STATES

## Abstract

The globalization of mosquito-borne arboviral diseases has placed more than half of the human population at risk. Understanding arbovirus ecology, including the role individual mosquito species play in virus transmission cycles, is critical for limiting disease. Canonical virus-vector groupings, such as *Aedes*- or *Culex*-associated flaviviruses, have historically been defined using virus detection in field-collected mosquitoes, mosquito feeding patterns, and vector competence, which quantifies the intrinsic ability of a mosquito to become infected with and transmit a virus during a subsequent blood feed. Herein, we quantitatively synthesize data from 68 laboratory-based vector competence studies of 111 mosquito-virus pairings of Australian mosquito species and viruses of public health concern to further substantiate existing canonical vector-virus groupings and quantify variation within these groupings. Our synthesis reinforces current canonical vector-virus groupings but reveals substantial variation within them. While *Aedes* species were generally the most competent vectors of canonical “*Aedes*-associated flaviviruses” (such as dengue, Zika, and yellow fever viruses), there are some notable exceptions; for example, *Aedes notoscriptus* is an incompetent vector of dengue viruses. *Culex* spp. were the most competent vectors of many traditionally *Culex*-associated flaviviruses including West Nile, Japanese encephalitis and Murray Valley encephalitis viruses, although some *Aedes* spp. are also moderately competent vectors of these viruses. Conversely, many different mosquito genera were associated with the transmission of the arthritogenic alphaviruses, Ross River, Barmah Forest, and chikungunya viruses. We also confirm that vector competence is impacted by multiple barriers to infection and transmission within the mesenteron and salivary glands of the mosquito. Although these barriers represent important bottlenecks, species that were susceptible to infection with a virus were often likely to transmit it. Importantly, this synthesis provides essential information on what species need to be targeted in mosquito control programs.

## Author summary

There are over 3,500 species of mosquitoes in the world, but only a small proportion are considered important vectors of arboviruses. Vector competence, the physiological ability of a mosquito to become infected with and transmit arboviruses, is used in combination with virus detection in field populations and analysis of vertebrate host feeding patterns to incriminate mosquito species in virus transmission cycles. Here, we quantified the vector competence of Australian mosquitoes for endemic and exotic viruses of public health concern by analyzing 68 laboratory studies of 111 mosquito-virus pairings. We found that Australia has species that could serve as efficient vectors for each virus tested and it is these species that should be targeted in control programs. We also corroborate previously identified virus-mosquito associations at the mosquito genus level but show that there is considerable variation in vector competence between species within a genus. We also confirmed that vector competence is influenced by infection barriers within the mosquito and the experimental protocols employed. The framework we developed could be used to synthesize vector competence experiments in other regions or expanded to a world-wide overview.

## Introduction

Mosquito-borne arboviruses infect hundreds of millions of humans and animals annually, thus representing a major global public and veterinary health threat [1,2]. The viruses responsible for severe clinical disease predominately belong to the viral families *Flaviviridae* and *Togaviridae* [2]. Globally important flaviviruses include the dengue viruses (DENVs) which cause 50 to 100 million symptomatic infections each year, the encephalitogenic Japanese encephalitis (JEV) and West Nile (WNV) viruses, which have expanded their geographical ranges in the last 25 years, and Zika virus (ZIKV), which is now recognized as a potential cause of severe congenital abnormalities [3–5]. Alphaviruses in the family *Togaviridae* from the Americas, such as eastern, western and Venezuelan encephalitis viruses are associated with encephalitis, whilst chikungunya (CHIKV) and Ross River (RRV) viruses, which originated in Africa and Australasia, respectively, have caused explosive epidemics of debilitating polyarthritis as they have invaded virgin ecosystems [6].

Combating the impact of arboviruses on human and animal health begins with understanding which mosquito species can transmit these viruses and how effectively they do so. Criteria proposed to incriminate mosquitoes as vectors of arboviruses include detection of viruses in mosquitoes in the field, temporal and spatial overlap with vertebrate hosts of the virus, blood feeding behavior that brings them into contact with these vertebrate hosts and the physiological ability of a mosquito species to become infected with and transmit a virus, known as its vector competence [7–9]. Phylogenetic analyses of the main arbovirus families have confirmed vector-virus associations and added evolutionary context to the links between mosquito genera and virus groups. For example, analysis of flavivirus non-structural five (NS5) gene sequences coupled with their respective vector associations initially classified the members of this family into three clusters: viruses without a known vector, tick-borne viruses, and mosquito-borne viruses [10]. Further analyses by Gaunt et al. [11] and Moureau et al. [12] using additional genes and more comprehensive datasets subdivided the mosquito-borne flaviviruses into *Aedes*-associated flaviviruses (AAFVs; e.g., DENVs in *Ae*. *aegypti* [13]) and *Culex*-associated flaviviruses (CAFVs; e.g., WNV in members of the *Culex pipiens* species complex [14]). In contrast to virus-mosquito associations observed in the flaviviruses, individual alphavirus species appear to utilize a wide range of mosquito genera as vectors [15].

**Fig 1:**
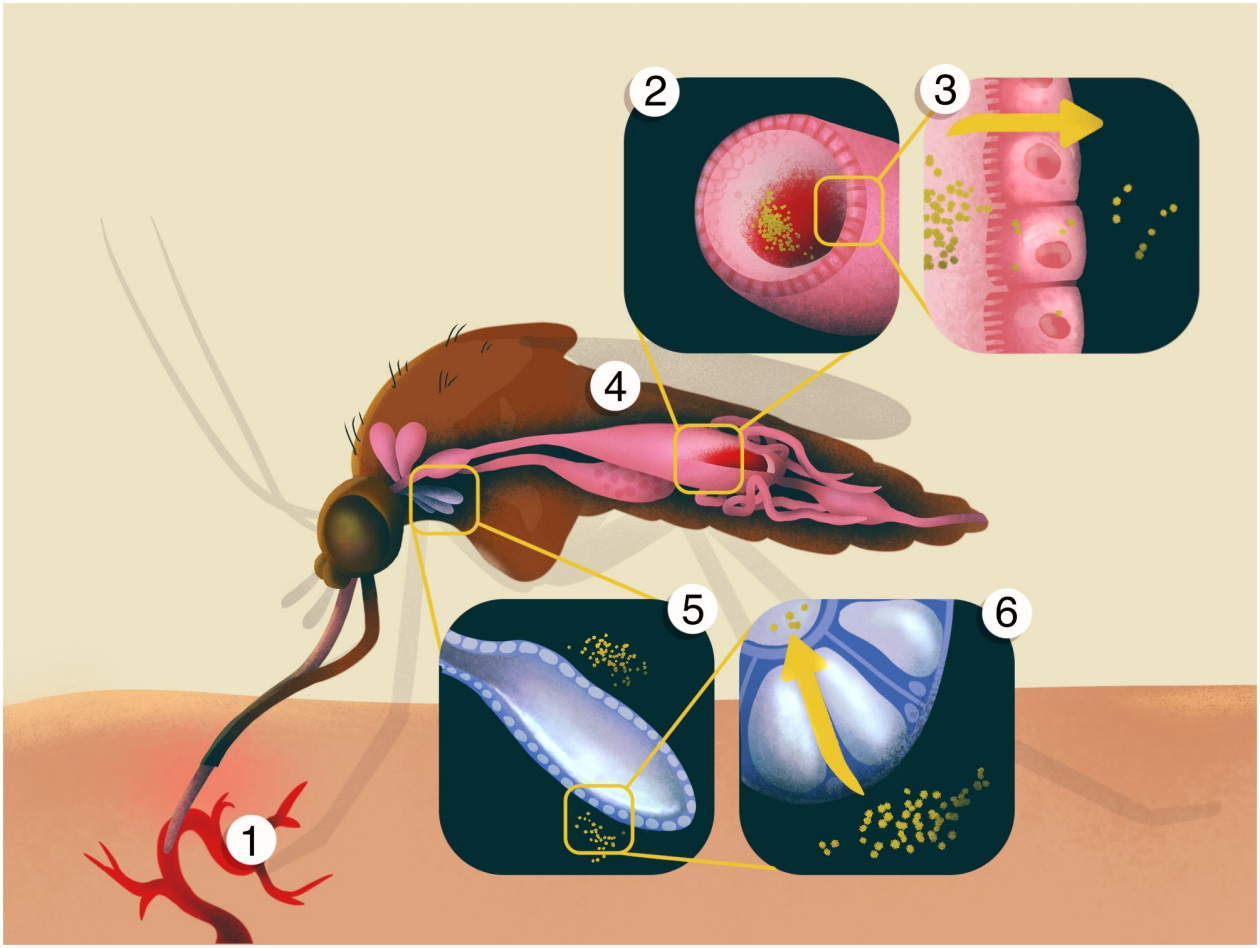
The steps involved in the successful transmission of an arbovirus by a mosquito. 1. Virus is ingested along with the blood meal from a viremic host. 2. The infectious blood meal is deposited in the posterior section of the mesenteron (midgut). 3. The virus infects and replicates in the epithelial cells of the mesenteron. 4. The virus disseminates from the mesenteronal epithelial cells via the hemolymph and infects other tissues, such as fat bodies or neural tissue, where it can undergo another round of replication. 5. The virus then infects the cells of the salivary glands. 6. The virus is then released in the saliva when the mosquito probes another vertebrate host.

Laboratory-based vector competence studies provide quantitative measures of a mosquito’s physiological ability to become infected with and transmit arboviruses by assessing the progression of a pathogen through four different barriers primarily associated with the mosquito mesenteron (midgut) and salivary glands (Fig 1), all of which must be overcome for the virus to ultimately be transmitted to a susceptible host [8,16,17]. After a mosquito takes a virus-laden blood meal (Fig 1, part 1), the virus must first overcome the mosquito’s mesenteronal infection barrier by binding to specific receptors on the epithelial cells (Fig 1, part 2). Passage of this barrier is measured by detecting virus in an infected mosquito’s body or the dissected mesenteron. The pathogen must then bypass the mesenteronal escape barrier, which limits dissemination of the pathogen from the mesenteronal epithelial cells [18,19] (Fig 1, part 3). The detection of virus in the legs, wings or head of a mosquito indicates that the virus has disseminated from the mesenteron via the hemolymph to other tissues and organs (Fig 1, part 4; [20]). Finally, the virus must successfully travel to, infect and replicate within the salivary glands to a level sufficient for transmission [8,21–23] (Fig 1, parts 5–6). Alternatively, if the virus does not infect the salivary glands or is not released in the saliva during probing and/or feeding, then the mosquito is considered to possess salivary gland infection or escape barriers, respectively. Expression of one or more of these barriers ultimately limits the ability of a given mosquito to transmit a virus.

In vector competence experiments, mosquitoes are exposed to either a previously infected animal or an artificial blood meal containing the virus [24]. At different time points post-exposure, mosquito infection and disseminated infection status is tested by processing whole bodies or body parts, such as mesenterons, heads, legs and/or wings. Mosquitoes are then assessed for their ability to transmit the pathogen using either a susceptible animal model [20,25] or an *in vitro* system of saliva collection [26]. Mosquitoes that do not express barriers to infection, dissemination, and transmission from the salivary glands are considered competent vectors. Conversely, species that strongly express any barriers that limit transmission are considered either poorly competent or incompetent; species whose mesenterons do not get infected after being exposed to an infectious blood meal are refractory to infection.

As single experiments tend to focus on the competence of a specific mosquito species for a specific virus, a synthesis of many vector competence experiments is needed to corroborate known and quantify variation within virus–mosquito associations across a broad range of mosquito and virus species. Synthesizing data across studies not only confirms the competence of established vectors but helps incriminate potential vectors of viruses for which we have no other information, such as exotic viruses which may have never encountered local mosquitoes in the field. Using data and measures of vector competence from 68 publications on Australian mosquito species, this study broadly assesses patterns of vector competence between mosquito taxa and medically important viruses of the *Flaviviridae* and *Togaviridae* families. The Australian vector competence studies provide the ideal data for us to address three primary sets of questions:

1. **Corroboration of mosquito–virus associations**: Which mosquito genera are most susceptible to infection and able to efficiently transmit each viral group? Do these mosquito–virus associations based on laboratory-based vector competence experiments reinforce or refute canonical associations based on virus isolation studies, analysis of host feeding patterns and even virus-vector phylogenetic relationships? Further, how much variation exists among mosquito species within a genus in their vector competence for the viruses within a given group?
2. **Influence of experimental methods and paucity of data on the outcomes of vector competence experiments**: What implications do the experimental design, methodology and extensiveness of individual studies have on interpreting the outcomes of vector competence experiments? Further, what missing data should be prioritized in future studies?
3. **Impact of intrinsic barriers to infection and transmission on vector competence**: Which barrier is the most limiting for vector competence? What is the strength of the correlation between infection, dissemination, and transmission?

Australia is an interesting case study for assessing vector competence because the island continent is climatically and biologically diverse, contains many ecologically distinct native and introduced mosquito species, and has experienced historical and emerging arbovirus threats. Australia has a history of outbreaks of endemic and exotic mosquito-borne arboviruses, the latter initiated by arrival of viremic travelers [27–30], and is potentially receptive to transmission of globally emergent arboviruses, such as ZIKV, CHIKV and the highly pathogenic North American WNV strains [31–33]. Thus, numerous laboratory-based experiments have been undertaken to assess the vector competence of Australian mosquito species for a range of virus species, with the overall aims of incriminating vectors and aiding targeted control strategies.

## Methods

### Data collection

All available Australian vector competence studies were collected by a single author (AvdH) using electronic databases, reference lists, hand searches, and institutional reports. A second author (EBS) then systematically searched for additional publications using a combination of the following search terms on Google Scholar: (mosquito* OR vector*) AND (competence OR infection OR dissemination OR transmission) AND (virus OR arbovirus OR flavivirus OR alphavirus) AND Australia. Studies were included if: a) they were original research; b) the viruses used were of human health importance; and c) they assessed at least one mosquito species with an Australian genetic background. These studies were then filtered to ensure that: a) viruses originated from field isolates and were not derived from infectious clones or recombinants (i.e., [34]); b) mosquitoes had not been modified so that virus replication was impacted (i.e., *Wolbachia* transinfected [35]); c) mosquitoes were exposed to virus by feeding on a viremic animal or an artificial infectious blood meal (i.e., not by intrathoracic inoculation [36], whereby the mesonteronal infection and escape barriers are bypassed); d) all mosquitoes were tested individually (i.e., transmission was not measured in batches); e) a minimum sample size of five mosquitoes at the experimental endpoint for each mosquito-vector pair; and f) transmission was measured via feeding on a suitable animal model or detection in saliva expectorates collected using *in vitro* saliva collection methods (as opposed to detection of virus in salivary glands which does not take into account the presence of a salivary gland escape barrier to transmission). In total, we gathered data from 68 publications, which included 389 individual experimental treatments (i.e., the exposure of a single cohort of mosquitoes of a species from a given origin to a unique strain of a given virus) on more than 25,000 total individual mosquitoes across 27 species spanning 6 genera and 13 viruses spanning 2 families (counting the four dengue virus serotypes separately) (Table 1). All data are available in the S1 File. The following data were extracted from each study:

- Vector characteristics: Mosquito species; origin
- Arbovirus characteristics: Virus species; isolate or strain
- Study methods: Virus dose to which mosquitoes were exposed; unit of measure of viral titer of the infectious blood meal (such as plaque forming units (PFU), tissue culture infectious dose_50_ (TCID_50_) and suckling mouse intracerebral inoculation lethal dose_50_ (SMIC LD_50_) which were all converted to infectious units per milliliter (IU/mL) for the analysis); method of virus exposure; method used to demonstrate transmission; assay to detect evidence of infection and/or transmission; days post exposure (DPE) to virus that infection, dissemination, and transmission were assessed; temperature at which mosquitoes were maintained; total number of mosquitoes exposed to the virus that were tested for infection, dissemination, and transmission
- Study results: proportion of mosquitoes that were exposed to the virus that were positive for infection, dissemination, and transmission. We emphasize that due to destructive sampling the experimentally observed proportions of mosquitoes positive for infection, dissemination, and transmission are conditioned on exposure (i.e., transmission is not conditioned on those already testing positive for dissemination).

For each virus, infection data were available for at least three species of mosquitoes (median of 7.5 species per virus) and dissemination data for at least two mosquito species per virus (median of 4 species per virus). Whilst transmission by individual mosquitoes was not assessed for some viruses (i.e., dengue virus type 1 [DENV-1]), the median number of species tested for transmission capability per virus was higher than for both infection and dissemination (median of 9 species per virus).

**Table 1:** Arboviruses assessed in vector competence experiments involving Australian mosquito species and included in the analysis.

Virus family	Virus species	Geographical distribution	Disease syndrome^a^	Vertebrate hosts^b^	Associated mosquito genera^c^
*Flaviviridae*	Dengue types 1–4	Asia, Africa, South and Central America	Hemorrhagic fever	Humans, non-human primates	*Aedes*
	Yellow fever	Africa, South America	Hemorrhagic fever	Humans, non-human primates	*Aedes*, *Haemagogus*, *Sabethes*
	Zika	Africa, Asia, South and Central America	Congenital malformations, Guillain-Barré syndrome	Humans, non-human primates	*Aedes*
	Japanese encephalitis	Asia, Australasia	Encephalitis, meningitis	Birds, Pigs	*Culex*
	Murray Valley encephalitis	Australasia	Encephalitis	Birds	*Culex*
	West Nile^d^	Africa, Asia, Australasia, North and South America, Europe	Encephalitis, meningitis	Birds	*Culex*
*Togaviridae*	Barmah Forest	Australasia	Arthritis	Mammals, birds	*Aedes*, *Culex*, *Coquillettidia*, *Verrallina*
	Chikungunya	Africa, Asia, South and Central America	Arthritis	Humans, non-human primates	*Aedes*
	Ross River	Australasia	Arthritis	Marsupials, humans	*Aedes*, *Culex*, *Coquillettidia*, *Verrallina*

^a^Many arbovirus infections cause non-specific febrile illness; the syndromes presented are the severe manifestations of disease.

^b^These are the major vertebrate hosts associated with transmission.

^c^Association of mosquito genera with the virus is based on virus isolation from the field and interaction with the vertebrate host as based on blood feeding patterns

^d^Includes the highly pathogenic North American strain and the Kunjin subtype which occurs in Australasia.

### Corroboration of mosquito–virus associations

We statistically tested for differences in infection and transmission proportions among the mosquito genera in our data set (*Aedes*, *Anopheles*, *Coquillettidia*, *Culex*, *Mansonia*, and *Verrallina*) for the following virus groups: *Aedes*-associated flaviviruses (AAFVs; yellow fever [YFV], ZIKV, DENV 1–4); *Culex*-associated flaviviruses (CAFVs; JEV, West Nile New York strain [WNV_NY99_], West Nile Kunjin strain [WNV_KUN_], Murray Valley encephalitis [MVEV]); and arthritogenic alphaviruses (CHIKV, RRV and Barmah Forest virus [BFV]). To do so we fitted two Generalized Linear Mixed Effects Models (GLMMs), each with a Binomial error distribution, that used the detected proportion of mosquitoes infected or transmitting as the response variable and the number of exposed mosquitoes as weights. Both models included the following fixed effects: an interaction of mosquito genus and virus group, experimental dose, and the number of DPE that mosquitoes were tested for infection or transmission. The 15 total fixed effects in each model were estimated using our full dataset: 1188 and 476 data points for the infection and transmission models, respectively. Each model also included random intercepts for mosquito species as well as “study”, which was used to control for additional variation not captured by the included fixed effects (due to, for example, variation in laboratory techniques and conditions, as well as between mosquito and virus strains).

We fitted these regression models in a Bayesian context using maximum *a posteriori* estimation with the *bglmer* function in the R [37] package blme [38]. We used Bayesian methods so that we could specify priors on the fixed effects in order to obtain credible intervals (CI) in the presence of complete separation (groups with all zeros, e.g., no *Culex* mosquitoes transmitted AAFVs) [39]. Specifically, we assumed weak Gaussian priors with a mean of zero and a standard deviation of three [40,41]. We drew inference on differences in infection and transmission proportions among mosquito genera–virus group pairings using the 95% CI for the response of each mosquito genera–virus group pairing calculated at the overall mean infectious dose and a DPE one standard deviation (SD) above the mean. We used one SD above the mean for DPE to allow for a sufficiently long extrinsic incubation period for a more realistic measure of transmission potential. This is because transmission will not occur until the intrinsic barriers to infection have been overcome and even highly competent vectors will often not be transmitting the virus at early timepoints post exposure. Point estimates and CI were extracted from the fitted models using the function *emmeans* in the package *emmeans* [42]. For fitted value vs residual diagnostic plots showing little to no pattern for these models see S1 Fig.

For these statistical analyses we focused on infection and transmission because they are the critical components of vector competence and were historically measured most frequently in experiments. Quantifying dissemination of the virus is a relatively recent addition to measurements used to assess the vector competence of mosquitoes for arboviruses [20], so including this metric in our statistical testing may limit the inclusion of studies reported prior to its introduction.

### Influence of experimental methods and paucity of data on the outcomes of vector competence experiments

Comparing infection and transmission probability at a specific dose (here the mean) and DPE (here one SD above the mean) provides a very narrow view of the continuous variation in proportions across dose- and time-response curves. That is, using a single somewhat arbitrary dose and DPE to quantify competence fails to capture the full spectrum of conditions experienced by mosquitoes feeding on viremic vertebrate hosts in nature. In an ideal world, we would instead calculate average infection probability, for example, by taking the mean of (or integrating over) the full non-linear infection probability curve across dose while weighting each dose by the probability a mosquito would receive that dose during a blood meal in nature.

This is, however, an impractical approach for comparing competence across all mosquito-virus pairings for two primary reasons. First, estimating unique slopes over dose and DPE for each mosquito genus-virus group pairing (i.e., with a “mosquito genus * virus group * dose * DPE” interaction term) would require experimental measures across dose and DPE for all pairings, which do not exist for Australian mosquitoes. Although our regression models included main effects of dose and DPE (allowing us to broadly control for variation in dose and DPE among mosquito-virus pairs), sparse data in most mosquito genus-virus group pairings prevented us from fitting unique dose and DPE slopes. Second, the probability weighted distribution of doses of a specific virus that a specific mosquito species would encounter in nature would require extensive data on host viremic responses to that virus and mosquito biting preferences on all these hosts.

Yet, this approach can be practical for some well-studied mosquito-virus pairs (e.g., see [43,44]). We estimated time- and dose-dependent regression models for *Ae*. *aegypti* as a case study here in order to: 1) explore variation in the shapes of infection and transmission curves across viruses for a single mosquito species; and 2) highlight the differences between a point estimate and the full infection and transmission functions. We fitted the proportion of *Ae*. *aegypti* infected, disseminated, and transmitting using GLMMs similar to those used previously, but restricted them to use data from *Ae*. *aegypti* and using a Frequentist framework with the R package *lme4* [45]. The model for infection included an interaction between virus species and virus dose, a fixed effect for DPE, and a random intercept of “study” to control for variation among citations not captured by the included fixed effects. The models for both dissemination and transmission included an interaction between virus species and DPE, a fixed effect for virus dose, and a random intercept for citation. Confidence intervals for infected, disseminated, and transmission proportions across dose and DPE were obtained from these models using 500 parametric bootstraps using the *bootMer* function in *lme4*. For fitted value vs residual diagnostic plots showing little to no pattern for these models see S2 Fig.

### Impact of intrinsic barriers to infection and transmission on vector competence

For each unique mosquito species–virus species pairing we extracted the highest observed proportion of mosquitoes of the given species that became infected with the given virus after ingestion of an infectious blood meal, irrespective of the dose administered or the day tested. We similarly identified the highest observed proportion of mosquitoes that developed a disseminated infection and that transmitted the virus. For each mosquito-virus pair, these maximal measures captured the single highest proportion observed across all virus doses the mosquito species was exposed to and all the days on which the species was tested.

The maximum proportion for each mosquito-virus pair is useful for two important reasons. First, it allows for an assessment of whether barriers to infection and transmission are still expressed or are overcome at the highest virus titers the mosquitoes have experimentally been exposed to. We tested for the level of expression of each barrier by: 1) calculating the reduction in the proportion of mosquitoes in which each virus was able pass through successive barriers (e.g., of those successfully infected, what proportion developed a disseminated infection) using a sample size weighted average across all mosquito-virus pairs for each barrier; 2) estimating the overall relationship between the expression of each barrier across all mosquito-virus pairs using the correlation between the maximum detected proportions of mosquitoes infected and transmitting across all mosquito–virus pairs (with the R function *cor*.*test*). Second, using maxima facilitates these analyses because they convert a highly unbalanced dataset (more values for infection than transmission for most mosquito-virus pairs) into a balanced one (one value for each endpoint).

## Results

### Mosquito-virus associations

#### *Aedes*-associated flaviviruses

**Fig 2:**
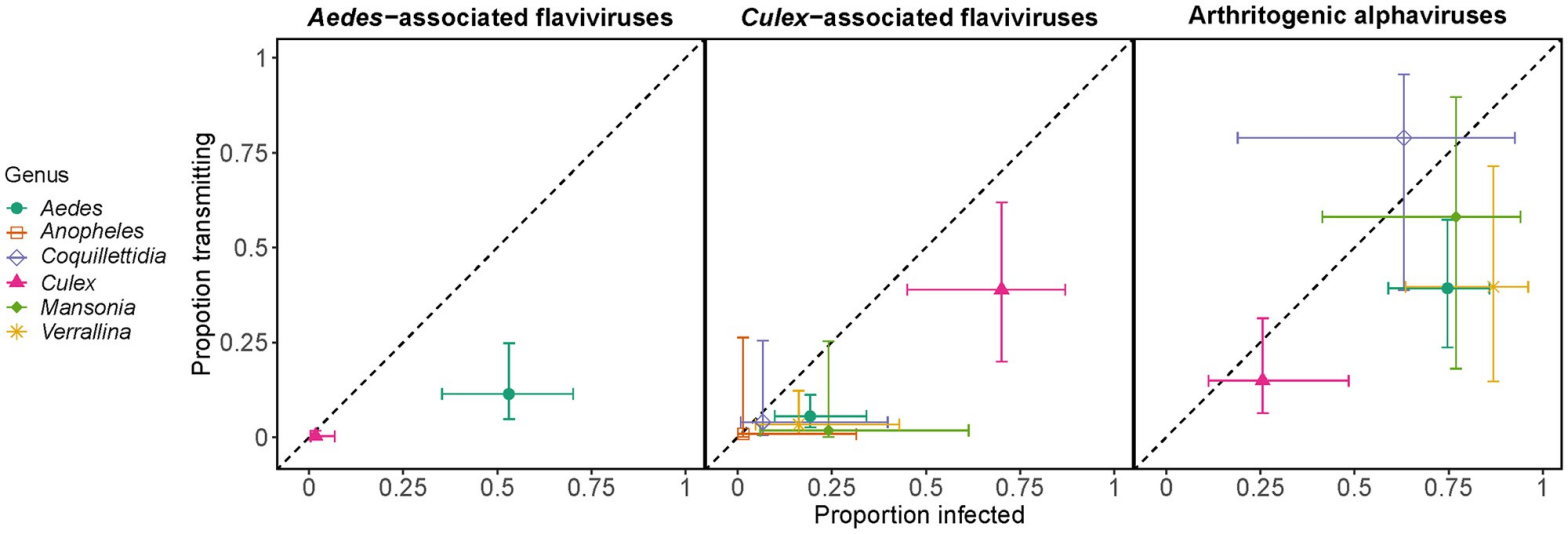
Estimated proportion of mosquitoes (grouped by genus: color) infected by (x-axis) and transmitting (y-axis) three virus groups (panels). Points show mean estimates and intervals show 95% credible intervals (CI) at the overall mean (across all experiments) dose and one SD above the overall mean DPE, obtained from the logistic regression models. Non-overlapping horizontal and vertical CI between two mosquito genera for a given viral group indicate statistically different infection and transmission capabilities, respectively, for those genera.

Only two Australian mosquito genera, *Aedes* and *Culex*, have been tested for their vector competence for AAFVs. A significantly larger proportion of *Aedes* individuals became infected with (mean: 53%; 95% CI: 35%-70%) and could transmit (mean: 11%; 95% CI: 4.8%-25%) these viruses than *Culex* individuals (infection: mean: 1.7%; 95% CI: 0.4%-6.8%; transmission: mean: 0.3%; 95% CI: 0.0%-1.7%) (Fig 2).

#### *Culex*-associated flaviviruses

A detectably higher proportion of *Culex* mosquitoes became infected with CAFVs (mean: 70%, 95% CI: 45%-87%) than all other genera apart from *Mansonia*; despite a large difference in their estimated means (Fig 2), the 95% CI of *Culex* and *Mansonia* overlapped due to small *Mansonia* sample sizes (22 total exposed mosquitoes) and highly variable responses among *Mansonia*-virus pairs (from 1/8 to 2/3). Significantly more *Culex* transmitted CAFVs (mean: 39%, 95% CI: 20%-62%) than *Aedes* and *Coquillettidia* (Fig 2); wide 95% CI for *Mansonia*, *Verrallina*, and *Anopheles* obscured our ability to statistically differentiate their transmission ability from that of *Culex* despite large differences in their estimated means (Fig 2). The CAFVs were the only group in the data set that contained at least one response from each mosquito genus for both infection and transmission, which strengthens the evidence for the importance of *Culex* as vectors of these viruses.

#### Arthritogenic alphaviruses

Overall, arthritogenic alphaviruses had the strongest ability to infect and be transmitted by multiple mosquito genera. A larger proportion of mosquitoes across all genera became infected with and transmitted arthritogenic alphaviruses than for CAFVs (difference in infection: β = 2.51, SE = 0.21, p < 0.05; transmission: β = 2.40, SE = 0.25, p < 0.05; coefficient on the logit-scale) and AAFVs (difference in infection: β = 0.95, SE = 0.16, p < 0.05; transmission: β = 1.64, SE = 0.47, p < 0.05; coefficient on the logit-scale).

Within the arthritogenic alphaviruses there were few clear differences among mosquito genera because of the high point estimates for both infection and transmission for all but *Culex*, but with wide CI. However, *Culex* is a clear outlier in that they have a much lower point estimate than all other genera for both infection and transmission and have non-overlapping CI with *Aedes* and *Verrallina* for infection, and *Coquillettidia* for transmission.

#### Variation within virus groups and mosquito genera

**Fig 3:**
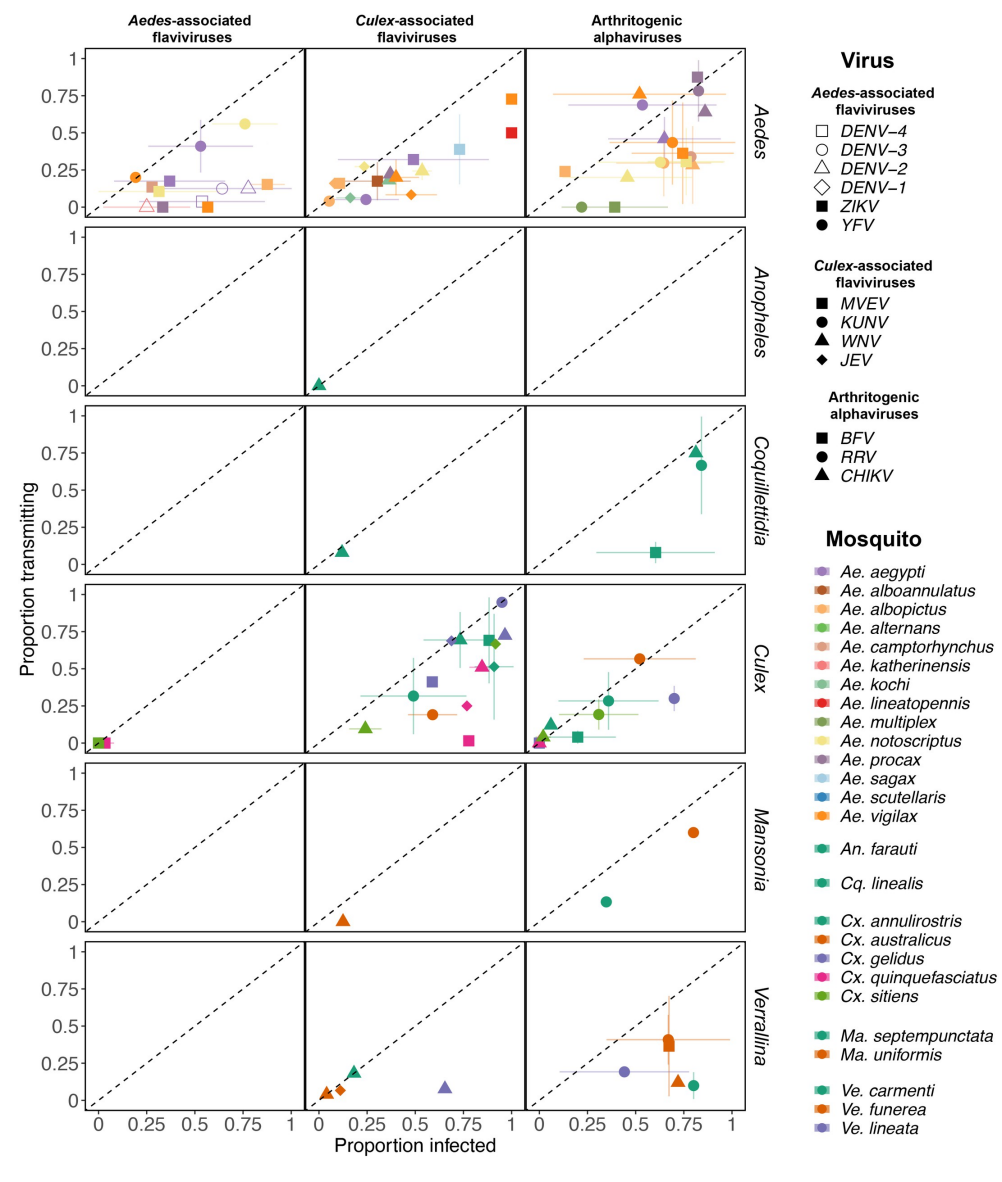
Raw proportion of mosquitoes (grouped by species: color and genus: rows) infected by (x-axis) and transmitting (y-axis) the viruses (shapes) separated by their viral grouping (columns). Each point shows the mean number of mosquitoes infected and transmitting across all experiments for that mosquito-virus pair; error bars show +/- one SD, calculated using the proportions across all experiments in that pair. Points without a horizontal or vertical error bar indicate pairs with only one experiment for infection or transmission, respectively. Mosquito-virus pairs without a measure for both infection and transmission are not shown (21 total pairs). Viruses are organized into three groups: *Aedes*-associated flaviviruses (yellow fever [YFV], Zika [ZIKV] and dengue [DENV, separated by serotype]); *Culex*-associated flaviviruses (Japanese encephalitis [JEV], West Nile New York strain [WNV], West Nile Kunjin strain [KUNV] and Murray Valley encephalitis [MVEV]); and arthritogenic alphaviruses (chikungunya [CHIKV], Ross River [RRV] and Barmah Forest [BFV]).

Mosquito species within a genus vary substantially in their ability to became infected with, disseminate, and transmit each of the virus species within each viral group. This variation can be viewed in one of two ways. First, the raw experimental proportions show that some individual mosquito-virus pairs have much higher infection and transmission proportions than others (Fig 3). Examples are numerous (Fig 3), but two that stand out are *Ae*. *aegypti* infected with BFV (19/501 positive across four experiments) vs *Ae*. *procax* infected with RRV (221/259 positive across five experiments) or *Cx*. *sitiens* vs *Cx*. *annulirostris* transmitting WNV_NY99_ (3/31 vs 53/77 transmitting respectively). While these two examples show stark contrasts, on the whole we caution against a direct interpretation of these raw data given that the SD error bars for a given mosquito-virus pair portrayed in Fig 3 consider just the variation in dose and DPE used in the experiments for that pair. As an alternative to considering all experimental results for all pairs, we also discuss variation in the maximum detected proportions for each mosquito-virus species pair as a measure of optimal potential in S1 Text and S3 and S4 Figs. Second, this variation can be quantified in a slightly different way using the estimated variation among species from the species-level random effect in the fitted GLMM; specifically, by comparing the species-level draws from this distribution (the individual deviates, also termed the conditional modes of the random effect). While these estimates cannot provide inference at the mosquito species–virus species level, they do provide a measure of each mosquito species’ overall vector competence across all viruses (S5 Fig). This figure shows, for example, that *Ae*. *procax* is broadly more susceptible to infection and more efficient at transmitting than *Ae*. *camptorhynchus*.

### Experimental design and paucity of data

Vector competence studies vary greatly in their experimental design, which can influence study outcomes. In particular, the experimental doses that mosquitoes were exposed to varied strongly both within and between viruses (S6 and S7 Figs). For example, only a single dose was used for WNV_NY99_ (6.7 log_10_ IU/mL), while doses spanned a wide range for other viruses, capturing more of the potential range of natural exposure to a viremic host (e.g., 3.2 to 10 log_10_ IU/mL for DENV-4 and 2.9 to 9.3 log_10_ IU/mL for RRV; S6 Fig). For species that were exposed to a range of virus doses, the dose that infected the maximum number of mosquitoes was generally among the higher of the doses that the mosquitoes were exposed to (S6 Fig). Similarly, the DPE on which mosquitoes were tested also varied strongly among studies (S8 and S9 Figs). Most notably, for some viruses (e.g., RRV, CHIKV) some studies tested for transmission continually from 2 DPE to around 15–20 DPE, whilst studies of other viruses used a very narrow window for assessing infection status and potential to transmit (e.g., WNV_NY99_, ZIKV).

As a case study, we used a GLMM to examine the continuous dose- and time-dependent responses of *Ae*. *aegypti*, which has been the subject of the most extensive assessment of vector competence of all Australian mosquito species. However, even for the best-studied pairings of *Ae*. *aegypti* exposed to CHIKV, ZIKV, and DENV-3, confidence intervals were wide mostly due to few data points and highly variable results at single doses and days across experiments (S10 Fig). Despite this uncertainty, these dose- and time-dependent continuous functions help to illustrate how relying on a single maximal competence measure could bias estimates of vector competence in practice, as it does not capture the conditions mosquitoes will experience when transmitting under natural conditions. However, until the substantial uncertainty is reduced, estimates from dose- and time-dependent continuous functions are unlikely to be more useful than a maximal measure.

### Barriers to infection and transmission

While these data made it difficult to quantitatively compare the impacts of each barrier (only 19% of all mosquito-virus pairings were assessed for infection, dissemination, and transmission, and only a small subset of these were assessed in the same laboratory setting), we obtained a coarse measure of the proportion of mosquitoes where the virus was unable to bypass each successive barrier using a sample size weighted average across all mosquito-virus pairs for each barrier. Across all mosquito-virus pairs and all experiments, conditional on exposure, 72% of mosquitoes became infected, 56% of mosquitoes disseminated virus, and 38% transmitted, implying that on average 72% of mosquitoes became infected, 77% of infected mosquitoes developed a disseminated infection, and 68% of mosquitoes with a disseminated infection went on to transmit. Although this method provides only a coarse measure of barrier importance relative to using single studies measuring infection, dissemination, and transmission for each mosquito species–virus pair [46], these results indicate that no single barrier stands out as the sole restriction to transmission, suggesting that all three are important.

Across all mosquito species and viruses, we found a strong positive correlation (r = 0.81, 95% confidence interval: 0.63–0.91) between mosquito species’ maxima for infection and transmission (S11 Fig). While some of this correlation can be attributed to research effort (mosquitoes studied more for infection are also studied more for transmission; S12 Fig), and detected maxima increase with increased sample sizes (p < 0.05, S13 Fig), the strength of this correlation points to the strong biological link between infection and transmission within mosquitoes. Specifically, mosquitoes that did not express a major mesenteronal infection barrier were more likely to transmit the virus.

## Discussion

Our analysis of vector competence experiments, involving 111 mosquito-virus pairings across 389 individual experimental treatments, quantified virus group–mosquito genus associations, corroborated existing canonical groupings, and refined our understanding of variation in the ability for different mosquito species to become infected with and transmit different arboviruses. Our synthesis reinforced the canonical groupings of *Aedes-* and *Culex-*associated flaviviruses; *Aedes* spp. were more competent than *Culex* spp. for AAFVs (the only two genera tested in our data set), whilst *Culex* spp. had the highest infection and transmission potential for CAFVs of all six genera tested (Fig 2). The association of CAFVs with *Culex* spp. in the Australian context is supported by field studies that have detected the majority of CAFVs in *Culex* spp., particularly *Cx*. *annulirostris* [47–49]. Though AAFVs are not endemic to Australia, a few instances of field evidence of AAFV carriage by Australian mosquitoes provide some independent support for our vector competence-based *Aedes-*flaviviruses associations. For example, DENV-2 RNA was detected in *Ae*. *aegypti* collected during a dengue outbreak in north Queensland in 2003 [50], and early experiments by Bancroft [51] and Cleland et al. [52] conducted during dengue outbreaks in the early 20^th^ century in Australia demonstrated that *Ae*. *aegypti* were infected with DENV as evidenced by transmission of the viruses to susceptible humans. Further, no *Cx*. *quinquefasciatus* tested by Cleland et al. [52] transmitted the virus, suggesting a low or negligible field infection rate and reinforcing the overall poor vector competence of members of the *Culex* genus for AAFVs. There have been no reported autochthonous cases of ZIKV or YFV in Australia.

In contrast to the flaviviruses, our analysis revealed that no single mosquito genus was clearly the most competent for infection with and transmission of the alphaviruses, which fits their previous description of vector promiscuity [15,53]. Broadly, *Culex* spp. had the lowest vector competence, *Coquillettidia* had the highest vector competence, and *Aedes* spp. had average susceptibility to infection and ability to transmit alphaviruses (Fig 2). These results concur with the plasticity in vector utilization observed for the arthritogenic alphaviruses [15] and the diversity of mosquitoes found infected with RRV and BFV in Australian mosquito populations [54–56]. Like the exotic flaviviruses, to date there has been no evidence of local transmission of CHIKV nor field isolation of this virus from Australian mosquitoes, despite the presence of populations of highly competent vectors (including *Ae*. *aegypti* and *Ae*. *albopictus*) in the state of Queensland and close geographical proximity of Australia to CHIKV endemic regions, such as Indonesia.

As vector competence tends to cluster by mosquito genus and virus sub-family (Fig 2), using mosquito genus and virus family-level information could help to constrain uncertainty about an unmeasured mosquito species-virus pair. This is potentially useful for predicting the consequences of an invading virus into a location with indigenous mosquito fauna not historically associated with the virus, or for the range expansion of a mosquito species that brings them into contact with circulating viruses in their new ecological niche. Whilst mosquito genus-level virus group associations were relatively robust, we did find substantial variation in vector competence at the mosquito species level (Figs 3, S3 and S4). Although many of the *Aedes* spp. established as global vectors of AAFVs had high competence for many AAFVs, we observed substantial variation in competence among *Aedes* species for individual AAFVs (Figs 3, S3 and S4). Commensurate with its role globally, Australian *Ae*. *aegypti* are highly susceptible to infection and readily transmit DENVs, YFV and ZIKV. In contrast, *Ae*. *notoscriptus*, which shares an overlapping ecological niche with *Ae*. *aegypti*, was an efficient laboratory vector of YFV, varied in response to challenge with different ZIKV strains, and was a poor vector of DENVs (Figs 3, S3 and S4). Although it could not be captured in our analysis, there is also considerable intraspecific variation in vector competence. For instance, *Cx*. *annulirostris* and *Ae*. *aegypti* collected from different locations in Australia significantly differed in their response to exposure to WNV [57,58] and DENVs [59], respectively. Conversely, there is also variation in vector competence of the mosquito species of the same origin for different virus strains, as evidenced by experiments with different DENV-3 and WNV isolates [30,60]. The degree of variation within each of the taxonomic levels highlights that although general virus–mosquito associations can help incriminate mosquitoes as vectors of arboviruses, location-specific experiments assessing the susceptibility and transmission ability of uncharacterized virus–mosquito pairing are still critical for establishing the role of a mosquito species in transmission cycles.

There are numerous intrinsic and interacting viral and vector traits that could determine the specificity or promiscuity of arbovirus-mosquito associations. Amino acid changes, even as a result of a single point mutation in an envelope protein, can facilitate virus adaptation to local mosquito vectors increasing the efficiency of their transmission [61–63]. While mutations that give rise to virus adaptation have the capacity to occur through a single passage in the mosquito [64], they are more likely to arise after many transmission generations. We found multiple instances where viruses were efficiently transmitted by a mosquito species they have never encountered in the field, a situation exemplified by the high vector competence of *Ae*. *vigilax* and *Ae*. *procax* for CHIKV, and of *Ae*. *notoscriptus* for YFV. This indicates that viral mutations and adaptation to local mosquito species are not necessarily a prerequisite for transmission by mosquito species the virus has not encountered. This supports a study in Thailand that found no evidence of adaptation of sympatric or allopatric isolates of DENVs to local *Ae*. *aegypti* populations [65]. It also indicates that some molecular determinants of vector competence may be conserved within mosquito and/or virus taxa, while others may be more specific. For example, *Aedes* spp. potentially possess a receptor or multiple receptors on the mesenteronal epithelial cells to which the envelope glycoproteins on the surface of AAFVs bind, thus initiating infection of the mesenteron [16]. In contrast, *Culex* spp. may not possess these specific receptors (or they may be restricted in density or diversity), limiting the number of binding sites for this group of viruses. Other intrinsic factors that influence virus–vector interactions are linked to diversity in the mosquito microbiome [66] and virome [67], and antiviral mechanisms in the mosquito [68]. Any of these factors could not only explain the virus-mosquito associations described above, but also how viruses that invade a virgin ecosystem are able to exploit the resident mosquito population, as occurred with WNV in North America [14] and establishment of YFV in *Haemagogus*/*Sabethes*-driven sylvatic cycles in South America [69].

We also found considerable variation in experimental conditions among the vector competence studies; mosquito and virus genotype, temperature, virus dose (S6 and S7 Figs), the DPE on which mosquitoes were tested (S8 and S9 Figs), and the nutritional status of the mosquito are just a few of the many factors that varied among studies that have been known to strongly influence the outcomes of individual mosquito-virus pairings [8, 24]. Further, experimental methods, such as the feeding protocol used to expose mosquitoes to the virus (e.g., live animal, membrane or blood-soaked pledget), virus isolate and passage used, origin of mosquito, mosquito laboratory generation number, and method used to assess transmission or assay to detect the presence of virus varied greatly among studies. Irrespective of this variation in experimental conditions, the central methodology of exposing mosquitoes to virus, maintaining them post exposure, and assessing their infection status and ability to transmit the virus was the same across studies.

On one hand, such variation makes it more difficult to predict the realized ability of a given mosquito to transmit a given virus and likely added much experimental uncertainty to the intrinsic variation within mosquito genera-virus family groupings. On the other hand, identifying the conditions a species requires to reach maximal transmission potential requires variation between and within experiments given the vast amount of variation that occurs during a transmission cycle under natural conditions. That is, when mosquitoes have been infected with only a single dose, and measured at the same time points (e.g., see WNV_NY99_ in S6–S9 Figs), we are limited to an understanding of which species are more competent under a narrowly-bounded setting and not the range of conditions that a mosquito would experience in nature when biting viremic hosts. By contrast, when mosquitoes are exposed to a wide range of doses and measured on many different DPE (e.g., see RRV in S6–S9 Figs), we are better able to capture variation in maxima among species and can take steps to quantify the full dose- and time-dependency of infection and transmission. It is important to determine this dose dependency because there is a threshold virus titer that the mosquito must imbibe in a blood meal to become infected, and it is generally accepted that highly competent vectors require a lower titer of virus than less competent vectors to become infected. Similarly, the time post exposure that mosquitoes transmit the virus is critical; the sooner that transmission occurs, the higher the proportion of mosquitoes that will survive the EIP and thus increase their status as a vector. A pertinent example of dose and time dependency was observed in *Ae*. *albopictus*, which not only was highly susceptible to CHIKV strains containing an alanine to valine mutation in the E1 envelope glycoprotein [63], but it was able to transmit it sooner than viruses without the mutation [70].

Dose-and time-dependent infection and transmission functions that utilize the full range of experimental conditions are powerful tools for quantifying the role different mosquito species play in viral transmission cycles. Specifically, when these continuous functions are combined with additional data, such as host viremia, mosquito feeding patterns, and/or climate conditions favorable to mosquito longevity, we are best able to gain understanding of the circumstances that allow different species to have increased transmission potential (i.e., a higher vectorial capacity; see [44,71]). Regression models like those we used for *Ae*. *aegypti* (S10 Fig) can provide the required data but are useful for only very well studied mosquito-virus pairs as they are more data intensive than calculating a maxima. That being said, with sufficient data it may be advisable to seek an alternative to logistic regression, as such an approach assumes that at a given dose and DPE all mosquitoes will become infected and transmit (asymptote of one), which is, in reality an unrealistic assumption. Ultimately, using the maxima is necessary for most mosquito-virus pairs (for many of which there is little data) and despite collapsing much variation it enables rapid incrimination of mosquitoes as arbovirus vectors.

Given that there are over 3,500 species of mosquitoes in the world, and 320 species in Australia [72,73], the choice of which mosquito species to include in vector competence experiments is usually based on their association with the target virus in endemic regions, field detections, interaction with vertebrate hosts and/or relative abundance. This can lead to a focus on certain genera, or certain species within a genus, which can potentially bias the analysis and its interpretation. For instance, only two genera were included in our analysis of the AAFVs, whilst six genera were included for the CAFVs. A synthesis of vector competence experiments from around the world could provide data on additional genera to mitigate this inherent bias, and potentially reinforce and expand on the outcomes of the current study.

This synthesis of vector competence studies is a valuable tool for understanding the vector competence of mosquitoes for many of the medically important arboviruses. When combined with data on vector abundance and human contact patterns [74], this information implicates species which need to be specifically targeted in control programs. For instance, we have shown that Torres Strait island populations of the highly invasive mosquito, *Ae*. *albopictus* are susceptible to infection with all viruses it was exposed to and was able to transmit them, albeit with considerable variation between viruses. This reinforces the importance of surveillance and control activities aimed at preventing the establishment of this species in populated areas of the Australian mainland [75]. Importantly, providing the consolidated information on vector competence means that mosquito control personnel can save precious time when responding to an arbovirus disease incident as they do not have to consult individual studies of specific mosquito-virus pairings.

## Supporting information

S1 Fig Residuals vs Fitted values for our primary analysis. (PDF)

S2 Fig Residuals vs Fitted values for our *Aedes*-specific GLMM. (PDF)

S3 Fig Maximum proportion infected for each mosquito-virus pair. **A)** Maximum proportion of mosquitoes positive for infection across all mosquito species tested for each virus (median and 80% quantiles across all mosquitoes). **B)** Maximum proportion of each mosquito species found positive for infection among all of the laboratory experiments for each mosquito-virus pair (represented by color and the bold-face top number in each box). The number of experimental mosquitoes tested in the study[s] in which the maximum proportion was observed is displayed in square brackets (in the case of multiple experiments finding the same maximum, the sample sizes were summed). Viruses are organized into three groups: *Aedes*-associated flaviviruses (yellow fever [YFV], Zika [ZIKV] and dengue [DENV, separated by serotype]); *Culex*-associated flaviviruses (Japanese encephalitis [JEV], West Nile New York strain [WNV_NY99_], West Nile Kunjin strain [WNV_KUNV_] and Murray Valley encephalitis [MVEV]); and arthritogenic alphaviruses (chikungunya [CHIKV], Ross River [RRV] and Barmah Forest [BFV]). Vertical dotted lines separate mosquito genera, from left to right: *Aedes* [*Ae*], *Anopheles* [*An*], *Coquillettidia* [*Cq*], *Culex* [*Cx*], *Mansonia* [*Ma*] and *Verrallina* [*Ve*].
(PDF)

S4 Fig Maximum proportion transmitting for each mosquito-virus pair. **A)** Maximum proportion of mosquitoes positive for transmission across all mosquito species tested for each virus (median and 80% quantiles across all mosquitoes). **B)** Maximum proportion of each mosquito species found positive for transmission among all of the laboratory experiments for each mosquito-virus pair (represented by color and the bold-face top number in each box). The number of experimental mosquitoes tested in the study[s] in which the maximum proportion was observed is displayed in square brackets (in the case of multiple experiments finding the same maximum, the sample sizes were summed). Viruses are organized into three groups and mosquito species are grouped into genera as in Fig 2.
(PDF)

S5 Fig Conditional modes of the ‘Species’ level random effect. Conditional modes of the `Species’ level random effect (points); infection in black and transmission in red. Error bars, which give 95% CIs, were calculated from the fitted model using the conditional modes and conditional covariances of the `Species’ level random effect. These values give individual mosquito species’ unique adjustments to the overall estimated intercept; higher values indicate more successful infection and transmission. Species are arranged from top to bottom from highest to lowest point estimates for infection.
(PDF)

S6 Fig Variation in dose across infection experiments for all mosquito-virus pairs. Viral doses mosquitoes were exposed to across all laboratory experiments that measured infection. Blue points show experimental doses that did not lead to the highest detected proportion of infected mosquitoes; red points show the infectious dose[s] that resulted in the highest observed infected proportion.
(PDF)

S7 Fig Variation in dose across transmission experiments for all mosquito-virus pairs. Viral doses mosquitoes were exposed to across all laboratory experiments that measured transmission. Blue points show experimental doses that did not lead to the highest detected proportion of transmitting mosquitoes; red points show the infectious dose[s] that resulted in the highest observed transmitting proportion.
(PDF)

S8 Fig Variation in DPE across infection experiments for all mosquito-virus pairs. Days post viral exposure on which mosquitoes were tested for infection. Blue points show experimental days that did not lead to the highest detected proportion of infected mosquitoes; red points show the day[s] that resulted in the highest observed infected proportion.
(PDF)

S9 Fig Variation in DPE across transmission experiments for all mosquito-virus pairs. Days post viral exposure on which mosquitoes were tested for transmission. Blue points show experimental days that did not lead to the highest detected proportion of transmitting mosquitoes; red points show the day[s] that resulted in the highest observed transmitting proportion.
(PDF)

S10 Fig *Aedes aegypti* infection and transmission curves across dose and DPE. Continuous functions for mosquito infection over dose (top), and for dissemination and transmission over time (middle and bottom, respectively). The results pictured here are from a generalized linear mixed effects model (GLMM) using *Aedes aegypti* for all viruses they have been tested with in the lab; however, here we only show the three viruses for which more than one data point exists for infection, dissemination, and transmission. For infection (top), medians (solid lines) and 95% confidence intervals (grey bands) are estimated using twelve days post exposure; for dissemination (middle) and transmission (bottom) estimates are drawn for a dose of 7 log_10_ IU/mL.
(PDF)

S11 Fig Correlation between maxima for infection and transmission. Multiple points of the same color and shape within a viral group represent results from different viruses within that group. The dashed line is a 1:1 line; points beneath the line represent mosquito-virus pairings with a greater infection probability than transmission probability.
(PDF)

S12 Fig Correlation in sample sizes for infection and transmission. Mosquito species frequently measured in infection experiments (total sample size across all experiments; X axis) were also more frequently measured in transmission experiments (total sample size across all experiments; Y axis). Each point for a mosquito species represents a different virus.
(PDF)

S13 Fig Relationship between sample size, dose and proportions positive for infection, dissemination, and transmission. Increased research effort led to a higher maximum proportion for infection, dissemination, and transmission (logistic regression, p < 0.05). Increased dose does not lead to a detectable increase in maximum proportion (logistic regression, p > 0.05). Each point represents a single virus-species pair.
(PDF)

S1 Text Mosquito-virus associations using maxima; variation among virus group-mosquito genera. (PDF)

S1 File Australian mosquito vector competence source data. (XLSX)

S1 Code Code used for model development. (ZIP)

## References

[1] Ketkar H, Herman D, Wang P. Genetic determinants of the re-emergence of arboviral diseases. Viruses. 2019;11: 150. doi: 10.3390/v11020150 30759739 PMC6410223

[2] Weaver SC, Charlier C, Vasilakis N, Lecuit M. Zika, chikungunya, and other emerging vector-borne viral diseases. Annu Rev Med. 2018;69: 395–408. doi: 10.1146/annurev-med-050715-105122 28846489 PMC6343128

[3] Mackenzie JS, Gubler DJ, Petersen LR. Emerging flaviviruses: the spread and resurgence of Japanese encephalitis, West Nile and dengue viruses. Nat Med. 2004;10: S98–S109. doi: 10.1038/nm1144 15577938

[4] Rasmussen SA, Jamieson DJ, Honein MA, Petersen LR. Zika virus and birth defects—reviewing the evidence for causality. N Engl J Med. 2016;374: 1981–1987. doi: 10.1056/NEJMsr1604338 27074377

[5] Stanaway JD, Shepard DS, Undurraga EA, Halasa YA, Coffeng LE, Brady OJ, et al. The global burden of dengue: an analysis from the Global Burden of Disease Study 2013. Lancet Infect Dis. 2016;16: 712–723. doi: 10.1016/S1473-3099(16)00026-8 26874619 PMC5012511

[6] Azar SR, Campos RK, Bergren NA, Camargos VN, Rossi SL. Epidemic alphaviruses: ecology, emergence and outbreaks. Microorganisms. 2020;8: 1167. 10.3390/microorganisms8081167 PMC7464724 32752150

[7] Barnett HC. The incrimination of arthropods as vectors of disease. 11th International Congress of Entomology. Vienna 1962; pp. 341–345.

[8] Hardy JL, Houk EJ, Kramer LD, Reeves WC. Intrinsic factors affecting vector competence of mosquitoes for arboviruses. Ann Rev Entomol. 1983;28: 229–262. doi: 10.1146/annurev.en.28.010183.001305 6131642

[9] Reeves WC. Arthropods as vectors and reservoirs of animal pathogenic viruses. In: Hallauer C, Meyer KF, editors. Handbuch der Virus Forschung. 4, Supplement 3. Vienna, Austria: Springer; 1957. pp. 177–202.

[10] Kuno G, Chang G-JJ, Tsuchiya KR, Karabatsos N, Cropp CB. Phylogeny of the Genus *Flavivirus*. J Virol. 1998;72: 73–83. 9420202 10.1128/jvi.72.1.73-83.1998 PMC109351

[11] Gaunt MW, Sall AA, de Lamballerie X, Falconar AK, Dzhivanian TI, Gould EA. Phylogenetic relationships of flaviviruses correlate with their epidemiology, disease association and biogeography. J Gen Virol. 2001;82: 1867–1876. doi: 10.1099/0022-1317-82-8-1867 11457992

[12] Moureau G, Cook S, Lemey P, Nougairede A, Forrester NL, Khasnatinov M, et al. New insights into flavivirus evolution, taxonomy and biogeographic history, extended by analysis of canonical and alternative coding sequences. PLoS One. 2015;10: e0117849. doi: 10.1371/journal.pone.0117849 25719412 PMC4342338

[13] Carrington LB, Simmons CP. Human to mosquito transmission of dengue viruses. Front Immunol. 2014;5: 290. doi: 10.3389/fimmu.2014.00290 24987394 PMC4060056

[14] Rochlin I, Faraji A, Healy K, Andreadis TG. West Nile virus mosquito vectors in North America. J Med Entomol. 2019;56: 1475–1490. doi: 10.1093/jme/tjz146 31549725

[15] Hanley KA, Weaver SC. Arbovirus evolution. In: Domingo E, Parrish CR, Holland JJ, editors. Origin and Evolution of Viruses. London: Academic Press; 2008. pp. 351–92.

[16] Franz AW, Kantor AM, Passarelli AL, Clem RJ. Tissue barriers to arbovirus infection in mosquitoes. Viruses. 2015;7: 3741–3767. doi: 10.3390/v7072795 26184281 PMC4517124

[17] Hardy JL. Susceptibility and resistance of vector mosquitoes. In: Monath TP, editor. The Arboviruses: Epidemiology and Ecology. I. Boca Raton, Florida: CRC Press; 1988. pp. 87–126.

[18] Kramer LD, Hardy JL, Houk EJ, Presser SB. Characterization of the mesenteronal infection with western equine encephalomyelitis virus in an incompetent strain of *Culex tarsalis*. Am J Trop Med Hyg. 1989;41: 241–250. 2774065 10.4269/ajtmh.1989.41.241

[19] Kramer LD, Hardy JL, Presser SB, Houk EJ. Dissemination barriers for western equine encephalomyelitis virus in *Culex tarsalis* infected after ingestion of low viral doses. Am J Trop Med Hyg 1981;30: 190–197. 7212166 10.4269/ajtmh.1981.30.190

[20] Turell MJ, Gargan TP II, Bailey CL. Replication and dissemination of Rift Valley fever virus in *Culex pipiens*. Am J Trop Med Hyg 1984;33: 176–181. 6696176 10.4269/ajtmh.1984.33.176

[21] Kramer LD, Ebel GD. Dynamics of flavivirus infection in mosquitoes. Adv Virus Res. 2003;60: 187–232. doi: 10.1016/s0065-3527(03)60006-0 14689695

[22] Mellor PS. Replication of arboviruses in insect vectors. J Comp Pathol. 2000;123: 231–247. doi: 10.1053/jcpa.2000.0434 11041993

[23] Richards SL, Anderson SL, Lord CC, Smartt CT, Tabachnick WJ. Relationships between infection, dissemination, and transmission of West Nile virus RNA in *Culex pipiens quinquefasciatus* (Diptera: Culicidae). J Med Entomol. 2012;49: 132–142. 22308781 10.1603/me10280 PMC3320798

[24] Azar SR, Weaver SC. Vector competence: what has Zika virus taught us? Viruses. 2019;11: 867. doi: 10.3390/v11090867 31533267 PMC6784050

[25] Kay B. Three modes of transmission of Ross River virus by *Aedes vigilax* (Skuse). Aust J Exp Biol Med Sci. 1982;60: 339–344. 6291499 10.1038/icb.1982.37

[26] Aitken THG. An in vitro feeding technique for artificially demonstrating virus transmission by mosquitoes. Mosquito News. 1977;37: 130–133.

[27] Bennett NM. Murray Valley encephalitis, 1974. Clinical features. Med J Aust. 1976;2: 446–450. 994930 10.5694/j.1326-5377.1976.tb130324.x

[28] Hanna JN, Ritchie SA, Phillips DA, Shield J, Bailey MC, Mackenzie JS, et al. An outbreak of Japanese encephalitis in the Torres Strait, Australia, 1995. Med J Aust. 1996;165: 256–260. doi: 10.5694/j.1326-5377.1996.tb124960.x 8816682

[29] Jansen CC, Shivas MA, May FJ, Pyke AT, Onn MB, Lodo K, et al. Epidemiologic, entomologic, and virologic factors of the 2014–15 Ross River virus outbreak, Queensland, Australia. Emerg Infect Dis. 2019;25: 2243–2252. doi: 10.3201/eid2512.181810 31742522 PMC6874252

[30] Ritchie SA, Pyke AT, Hall-Mendelin S, Day A, Mores CN, Christofferson RC, et al. An explosive epidemic of DENV-3 in Cairns, Australia. PLoS One. 2013;8: e68137. doi: 10.1371/journal.pone.0068137 23874522 PMC3712959

[31] Jansen CC, Ritchie SA, van den Hurk AF. The role of Australian mosquito species in the transmission of endemic and exotic West Nile virus strains. Int J Environ Res Public Health. 2013;10: 3735–3752. doi: 10.3390/ijerph10083735 23965926 PMC3774466

[32] Viennet E, Knope K, Faddy HM, Williams CR, Harley D. Assessing the threat of chikungunya virus emergence in Australia. Commun Dis Intell. 2013;37: E136–143. 24168087 10.33321/cdi.2013.37.19

[33] Viennet E, Mincham G, Frentiu FD, Jansen CC, Montgomery BL, Harley D, et al. Epidemic potential for local transmission of Zika virus in 2015 and 2016 in Queensland, Australia. PLoS Curr. 2016;8. doi: 10.1371/currents.outbreaks.73d82b08998c6d729c41ef6cdcc80176 28123859 PMC5222544

[34] Melian EB, Hall-Mendelin S, Du F, Owens N, Bosco-Lauth AM, Nagasaki T, et al. Programmed ribosomal frameshift alters expression of West Nile virus genes and facilitates virus replication in birds and mosquitoes. PLoS Pathog. 2014;10: e1004447. doi: 10.1371/journal.ppat.1004447 25375107 PMC4223154

[35] Moreira LA, Iturbe-Ormaetxe I, Jeffery JA, Lu G, Pyke AT, Hedges LM, et al. A *Wolbachia* symbiont in *Aedes aegypti* limits infection with dengue, Chikungunya, and *Plasmodium*. Cell. 2009;139: 1268–1278. 20064373 10.1016/j.cell.2009.11.042

[36] McLean DM. Multiplication of viruses in mosquitoes following feeding and injection into the body cavity. Aust J Exp Biol Med Sci. 1955;33: 53–66. doi: 10.1038/icb.1955.7 14389174

[37] R Team. R: A language and environment for statistical computing. Vienna, Austria: R Foundation for Statistical Computing; 2021.

[38] Chung Y, Rabe-Hesketh S, Dorie V, Gelman A, Liu J. A nondegenerate penalized likelihood estimator for variance parameters in multilevel models. Psychometrika. 2013;78: 685–709. doi: 10.1007/s11336-013-9328-2 24092484

[39] Allison PD. Convergence Failures in Logistic Regression. SAS Global Forum 2008; 2008.

[40] Gelman A, Jakulin A, Pittau MG, Su YS. A weakly informative default prior distribution for logistic and other regression models. Ann Appl Stat. 2008;2: 1360–1383.

[41] Quiñones AE, Wcislo WT. Cryptic extended brood care in the facultatively eusocial sweat bee *Megalopta genalis*. Insectes Sociaux. 2015;62: 307–313. 26097252 10.1007/s00040-015-0409-3 PMC4469088

[42] Lenth R, Lenth MR. Package ‘lsmeans’. Am Stat. 2018;34: 216–221.

[43] Focks DA, Daniels E, Haile DG, Keesling JE. A simulation model of the epidemiology of urban dengue fever: literature analysis, model development, preliminary validation, and samples of simulation results. Am J Trop Med Hyg. 1995;53: 489–506. doi: 10.4269/ajtmh.1995.53.489 7485707

[44] Kain MP, Skinner EB, van den Hurk AF, McCallum H, Mordecai EA. Physiology and ecology combine to determine host and vector importance for Ross River virus. eLife. 2021;10: e67018. doi: 10.7554/eLife.67018 34414887 PMC8457839

[45] Bates D, Mächler M, Bolker B, Walker S. Fitting linear mixed-effects models using lme4. J Stat Soft. 2015;67: 1–48.

[46] Tesla B, Demakovsky LR, Packiam HS, Mordecai EA, Rodriguez AD, Bonds MH, et al. Estimating the effects of variation in viremia on mosquito susceptibility, infectiousness, and *R*_0_ of Zika in *Aedes aegypti*. PLoS Negl Trop Dis. 2018;12: e0006733. 30133450 10.1371/journal.pntd.0006733 PMC6122838

[47] Broom AK, Lindsay MD, Wright AE, Smith DW, Mackenzie JS. Epizootic activity of Murray Valley encephalitis and Kunjin viruses in an aboriginal community in the southeast Kimberley region of Western Australia: results of mosquito fauna and virus isolation studies. Am J Trop Med Hyg. 2003;69: 277–283. 14628944

[48] Doherty RL, Carley JG, Mackerras MJ, Marks EN. Studies of arthropod-borne virus infections in Queensland, III. Isolation and characterisation of virus strains from wild-caught mosquitoes in north Queensland. Aust J Exp Biol Med Sci. 1963;41: 17–40. 14028387 10.1038/icb.1963.2

[49] Ritchie SA, Phillips D, Broom A, Mackenzie J, Poidinger M, van den Hurk A. Isolation of Japanese encephalitis virus from *Culex annulirostris* in Australia. Am J Trop Med Hyg 1997;56: 80–84. 9063367 10.4269/ajtmh.1997.56.80

[50] Ritchie SA, Long S, Smith G, Pyke A, Knox TB. Entomological investigations in a focus of dengue transmission in Cairns, Queensland, Australia, by using the sticky ovitraps. J Med Entomol. 2004;41: 1–4. doi: 10.1603/0022-2585-41.1.1 14989339

[51] Bancroft TL. On the etiology of dengue fever. Aust Med Gaz. 1906;25: 17–8.

[52] Cleland JB, Bradley B, McDonald W. On the transmission of Australian dengue by the mosquito *Stegomyia fasciata*. Med J Aust. 1916;2: 179–84, 200–5.

[53] Coffey LL, Forrester N, Tsetsarkin K, Vasilakis N, Weaver SC. Factors shaping the adaptive landscape for arboviruses: implications for the emergence of disease. Future Microbiol. 2013;8: 155–176. doi: 10.2217/fmb.12.139 23374123 PMC3621119

[54] Harley D, Ritchie S, Phillips D, van den Hurk A. Mosquito isolates of Ross River virus from Cairns, Queensland, Australia. Am J Trop Med Hyg. 2000;62: 561–565. doi: 10.4269/ajtmh.2000.62.561 11289664

[55] Lindsay MD, Johansen CA, Smith DW, Wallace MJ, Mackenzie JS. An outbreak of Barmah Forest virus disease in the south-west of Western Australia. Med J Aust. 1995;162: 291–294. doi: 10.5694/j.1326-5377.1995.tb139902.x 7715489

[56] Ritchie SA, Fanning ID, Phillips DA, Standfast HA, McGinn D, Kay BH. Ross River virus in mosquitoes (Diptera: Culicidae) during the 1994 epidemic around Brisbane, Australia. J Med Entomol. 1997;34: 156–159. doi: 10.1093/jmedent/34.2.156 9103757

[57] Jansen CC, Webb CE, Northill JA, Ritchie SA, Russell RC, van den Hurk AF. Vector competence of Australian mosquito species for a North American strain of West Nile virus. Vector Borne Zoonotic Dis. 2008;8: 805–811. doi: 10.1089/vbz.2008.0037 18973445

[58] Kay BH, Fanning ID, Carley JG. The vector competence of Australian *Culex annulirostris* with Murray Valley encephalitis and Kunjin viruses. Aust J Exp Biol Med Sci. 1984;62: 641–650. 6100042 10.1038/icb.1984.61

[59] Knox TB, Kay BH, Hall RA, Ryan PA. Enhanced vector competence of *Aedes aegypti* (Diptera: Culicidae) from the Torres Strait compared with mainland Australia for dengue 2 and 4 viruses. J Med Entomol. 2003;40:950–956. 14765675 10.1603/0022-2585-40.6.950

[60] van den Hurk AF, Hall-Mendelin S, Webb CE, Tan CS, Frentiu FD, Prow NA, et al. Role of enhanced vector transmission of a new West Nile virus strain in an outbreak of equine disease in Australia in 2011. Parasit Vectors. 2014;7: 586. doi: 10.1186/s13071-014-0586-3 25499981 PMC4280035

[61] Anderson JR, Rico-Hesse R. *Aedes aegypti* vectorial capacity is determined by the infecting genotype of dengue virus. Am J Trop Med Hyg. 2006;75: 886–892. 17123982 PMC1993907

[62] Ebel GD, Carricaburu J, Young D, Bernard KA, Kramer LD. Genetic and phenotypic variation of West Nile virus in New York, 2000–2003. Am J Trop Med Hyg. 2004;71: 493–500. 15516648

[63] Tsetsarkin KA, Vanlandingham DL, McGee CE, Higgs S. A single mutation in chikungunya virus affects vector specificity and epidemic potential. PLoS Pathog. 2007;3: e201. doi: 10.1371/journal.ppat.0030201 18069894 PMC2134949

[64] Stapleford KA, Coffey LL, Lay S, Borderia AV, Duong V, Isakov O, et al. Emergence and transmission of arbovirus evolutionary intermediates with epidemic potential. Cell Host Microbe. 2014;15: 706–716. doi: 10.1016/j.chom.2014.05.008 24922573

[65] Fansiri T, Pongsiri A, Klungthong C, Ponlawat A, Thaisomboonsuk B, Jarman RG, et al. No evidence for local adaptation of dengue viruses to mosquito vector populations in Thailand. Evol Appl. 2016;9: 608–618. doi: 10.1111/eva.12360 27099625 PMC4831462

[66] Ramirez JL, Souza-Neto J, Torres Cosme R, Rovira J, Ortiz A, Pascale JM, et al. Reciprocal tripartite interactions between the *Aedes aegypti* midgut microbiota, innate immune system and dengue virus influences vector competence. PLoS Negl Trop Dis. 2012;6: e1561. doi: 10.1371/journal.pntd.0001561 22413032 PMC3295821

[67] Bolling BG, Olea-Popelka FJ, Eisen L, Moore CG, Blair CD. Transmission dynamics of an insect-specific flavivirus in a naturally infected *Culex pipiens* laboratory colony and effects of co-infection on vector competence for West Nile virus. Virology. 2012;427: 90–97. 22425062 10.1016/j.virol.2012.02.016 PMC3329802

[68] Xi Z, Ramirez JL, Dimopoulos G. The *Aedes aegypti* toll pathway controls dengue virus infection. PLoS Pathog. 2008;4: e1000098. 18604274 10.1371/journal.ppat.1000098 PMC2435278

[69] Silva NIO, Sacchetto L, de Rezende IM, Trindade GS, LaBeaud AD, de Thoisy B, et al. Recent sylvatic yellow fever virus transmission in Brazil: the news from an old disease. Virol J. 2020;17: 9. doi: 10.1186/s12985-019-1277-7 31973727 PMC6979359

[70] Dubrulle M, Mousson L, Moutailler S, Vazeille M, Failloux AB. Chikungunya virus and *Aedes* mosquitoes: saliva is infectious as soon as two days after oral infection. PLoS One. 2009;4: e5895. 19521520 10.1371/journal.pone.0005895 PMC2690823

[71] Jetten TH, Focks DA. Potential changes in the distribution of dengue transmission under climate warming. Am J Trop Med Hyg. 1997;57: 285–297. doi: 10.4269/ajtmh.1997.57.285 9311638

[72] Webb C, Doggett S, Russell R. A Guide to Mosquitoes of Australia. Canberra: CSIRO Publishing; 2016.

[73] Wilkerson RC, Linton Y-M, Strickman D. Mosquitoes of the World. Baltimore: Johns Hopkins University Press; 2021.

[74] Jansen CC, Williams CR, van den Hurk AF. The usual suspects: comparison of the relative roles of potential urban chikungunya virus vectors in Australia. PLoS One. 2015;10: e0134975. doi: 10.1371/journal.pone.0134975 26247366 PMC4527740

[75] van den Hurk AF, Nicholson J, Beebe NW, Davis J, Muzari OM, Russell RC, et al. Ten years of the Tiger: *Aedes albopictus* presence in Australia since its discovery in the Torres Strait in 2005. One Health. 2016;2: 19–24. 28616473 10.1016/j.onehlt.2016.02.001 PMC5462651

