# Supplementary material for: Not all mosquitoes are created equal: A synthesis of vector competence experiments reinforces virus associations of Australian mosquitoes
Source: PLoS Negl Trop Dis. 2022 Oct 4;16(10):e0010768. doi: 10.1371/journal.pntd.0010768 (PMC9565724; doi:10.1371/journal.pntd.0010768)
Supplement: S1 Fig — (PDF) [file pntd.0010768.s001.pdf]

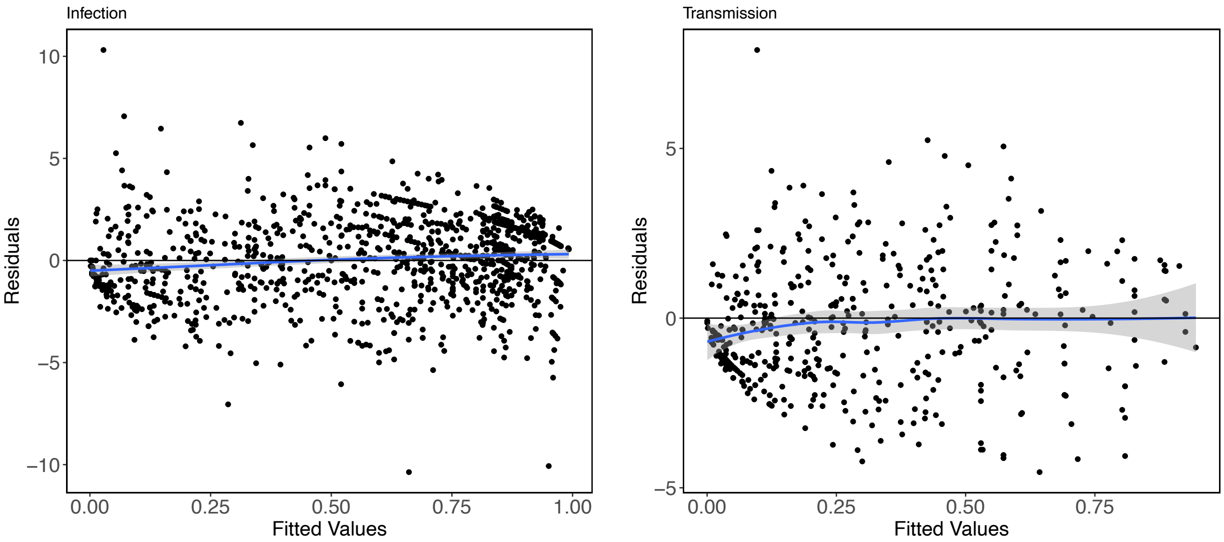

Figure S1. Residuals vs fitted values for the two Binomial GLMM models fit for our primary analysis.
