# Supplementary figures and images for: Not all mosquitoes are created equal: A synthesis of vector competence experiments reinforces virus associations of Australian mosquitoes
Source: PLoS Negl Trop Dis. 2022 Oct 4;16(10):e0010768. doi: 10.1371/journal.pntd.0010768 (PMC9565724; doi:10.1371/journal.pntd.0010768)

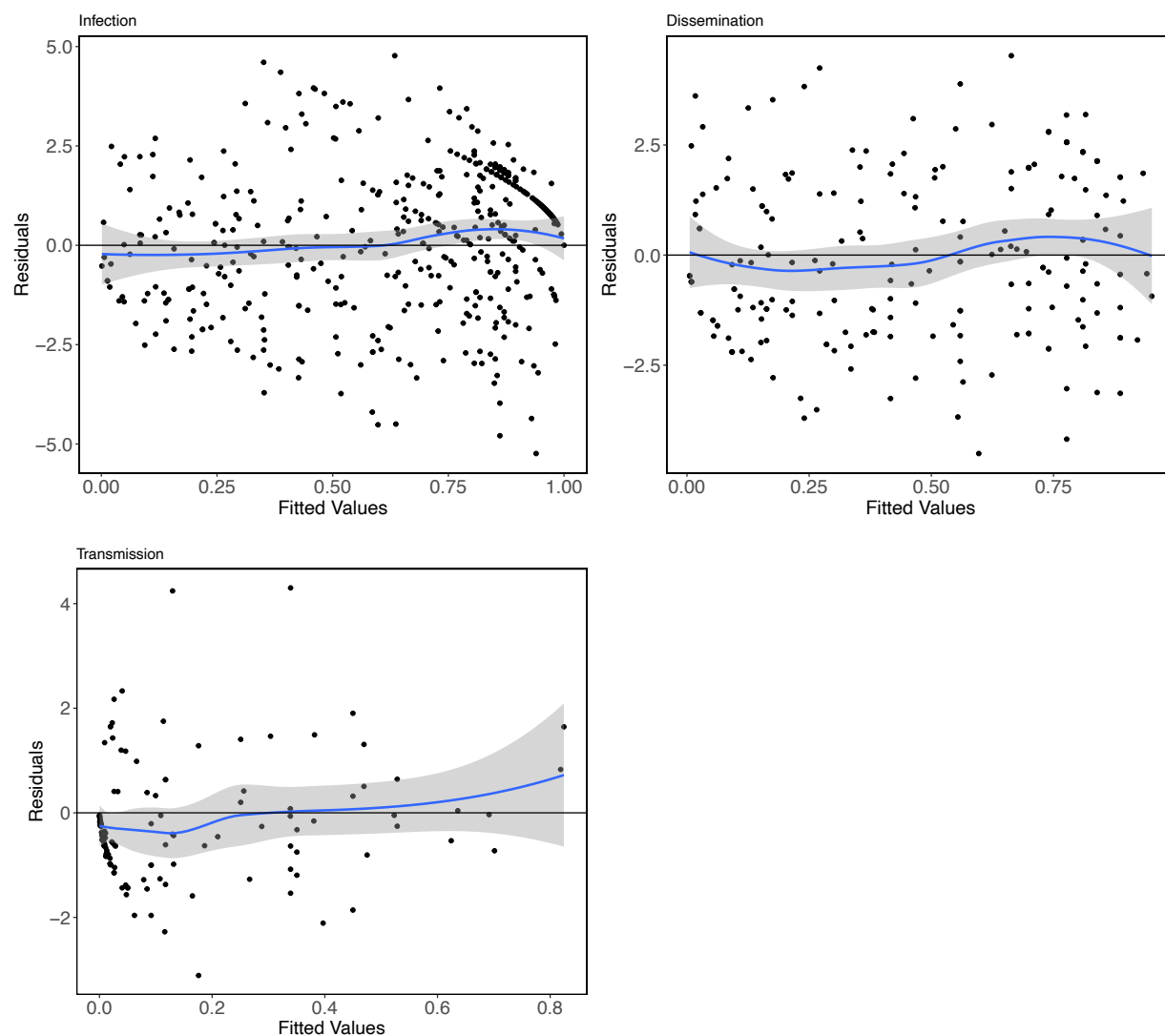

**Figure S2.** Residuals vs fitted values for the three Binomial GLMM models fit using *Aedes aegypti*.

Supplement: S2 Fig — (PDF) [file pntd.0010768.s002.pdf]
