# Supplementary material for: Not all mosquitoes are created equal: A synthesis of vector competence experiments reinforces virus associations of Australian mosquitoes
Source: PLoS Negl Trop Dis. 2022 Oct 4;16(10):e0010768. doi: 10.1371/journal.pntd.0010768 (PMC9565724; doi:10.1371/journal.pntd.0010768)
Supplement: S5 Fig — Conditional modes of the `Species’ level random effect (points); infection in black and transmission in red. Error bars, which give 95% CIs, were calculated from the fitted model using the conditional modes and conditional covariances of the `Species’ level random effect. These values give individual mosquito species’ unique adjustments to the overall estimated intercept; higher values indicate more successful infection and transmission. Species are arranged from top to bottom from highest to lowest point estimates for infection. (PDF) [file pntd.0010768.s005.pdf]

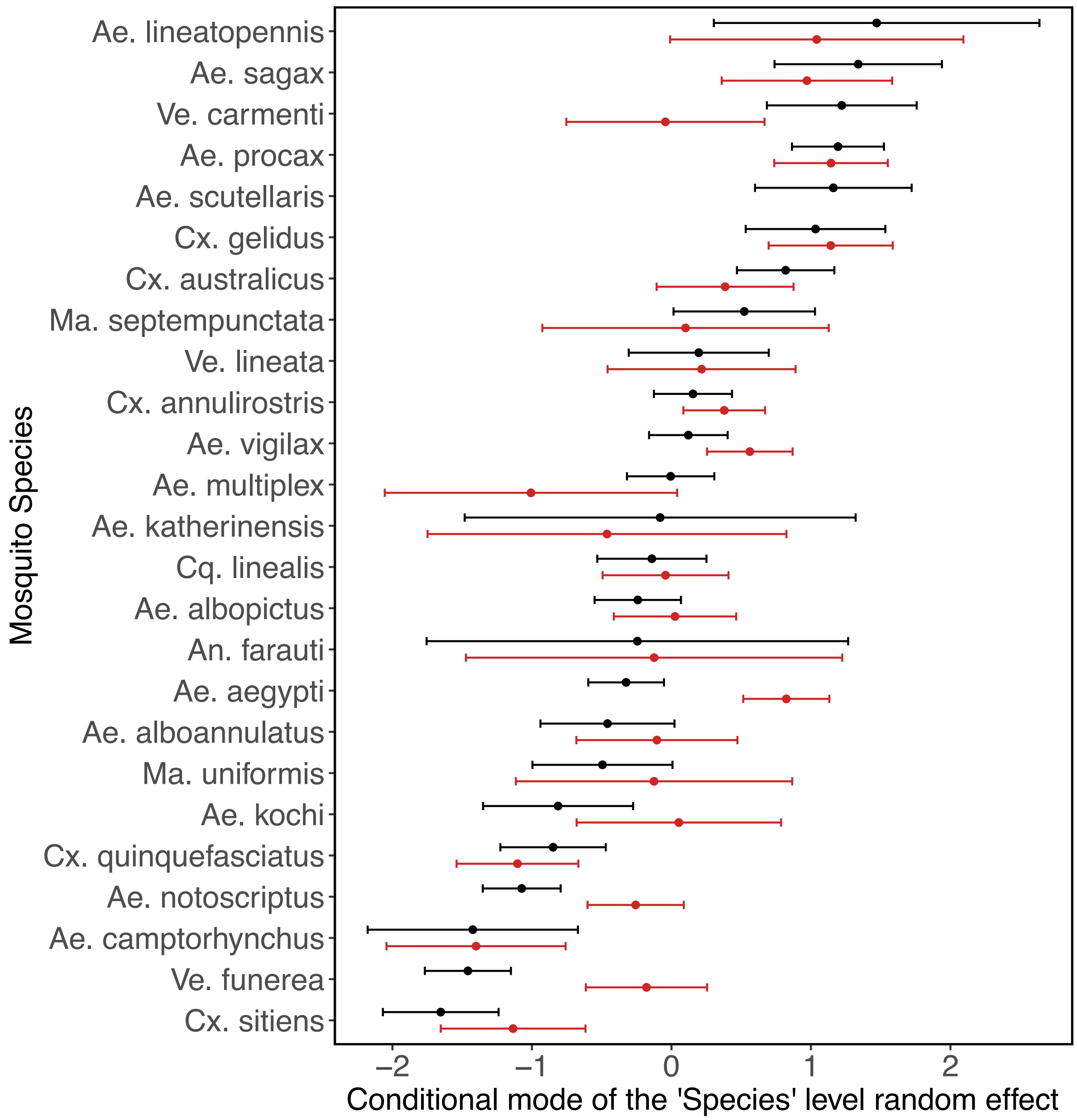

Figure S5. Conditional modes of the `Species' level random effect (points); infection in back, transmission in red. Error bars, which give 95% CIs, were calculated from the fitted model using the conditional modes and conditional covariances of the `Species' level random effect. These values give individual mosquito species' unique adjustments to the overall estimated intercept; higher values indicate more successful infection and transmission. Species are arranged from top to bottom from highest to lowest point estimates for infection.
