# Supplementary material for: Not all mosquitoes are created equal: A synthesis of vector competence experiments reinforces virus associations of Australian mosquitoes
Source: PLoS Negl Trop Dis. 2022 Oct 4;16(10):e0010768. doi: 10.1371/journal.pntd.0010768 (PMC9565724; doi:10.1371/journal.pntd.0010768)
Supplement: S7 Fig — Viral doses mosquitoes were exposed to across all laboratory experiments that measured transmission. Blue points show experimental doses that did not lead to the highest detected proportion of transmitting mosquitoes; red points show the infectious dose[s] that resulted in the highest observed transmitting proportion. (PDF) [file pntd.0010768.s007.pdf]

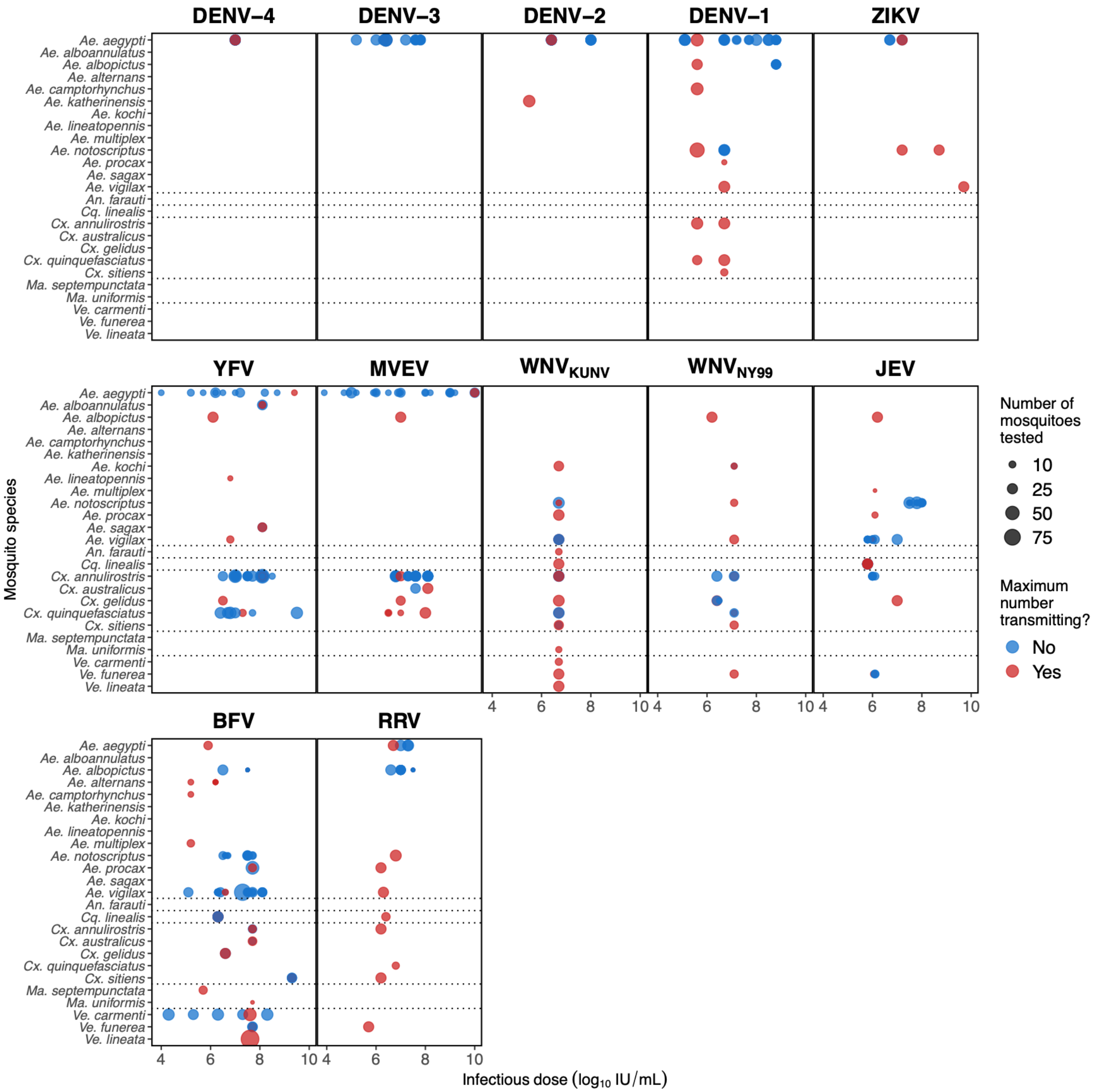

Figure S7. Viral doses mosquitoes were exposed to across all laboratory experiments that measured transmission. Blue points show experimental doses that did not lead to the highest detected proportion of transmitting mosquitoes; red points show the infectious dose[s] that resulted in the highest observed transmitting proportion.
