# Supplementary material for: Not all mosquitoes are created equal: A synthesis of vector competence experiments reinforces virus associations of Australian mosquitoes
Source: PLoS Negl Trop Dis. 2022 Oct 4;16(10):e0010768. doi: 10.1371/journal.pntd.0010768 (PMC9565724; doi:10.1371/journal.pntd.0010768)
Supplement: S8 Fig — Days post viral exposure on which mosquitoes were tested for infection. Blue points show experimental days that did not lead to the highest detected proportion of infected mosquitoes; red points show the day[s] that resulted in the highest observed infected proportion. (PDF) [file pntd.0010768.s008.pdf]

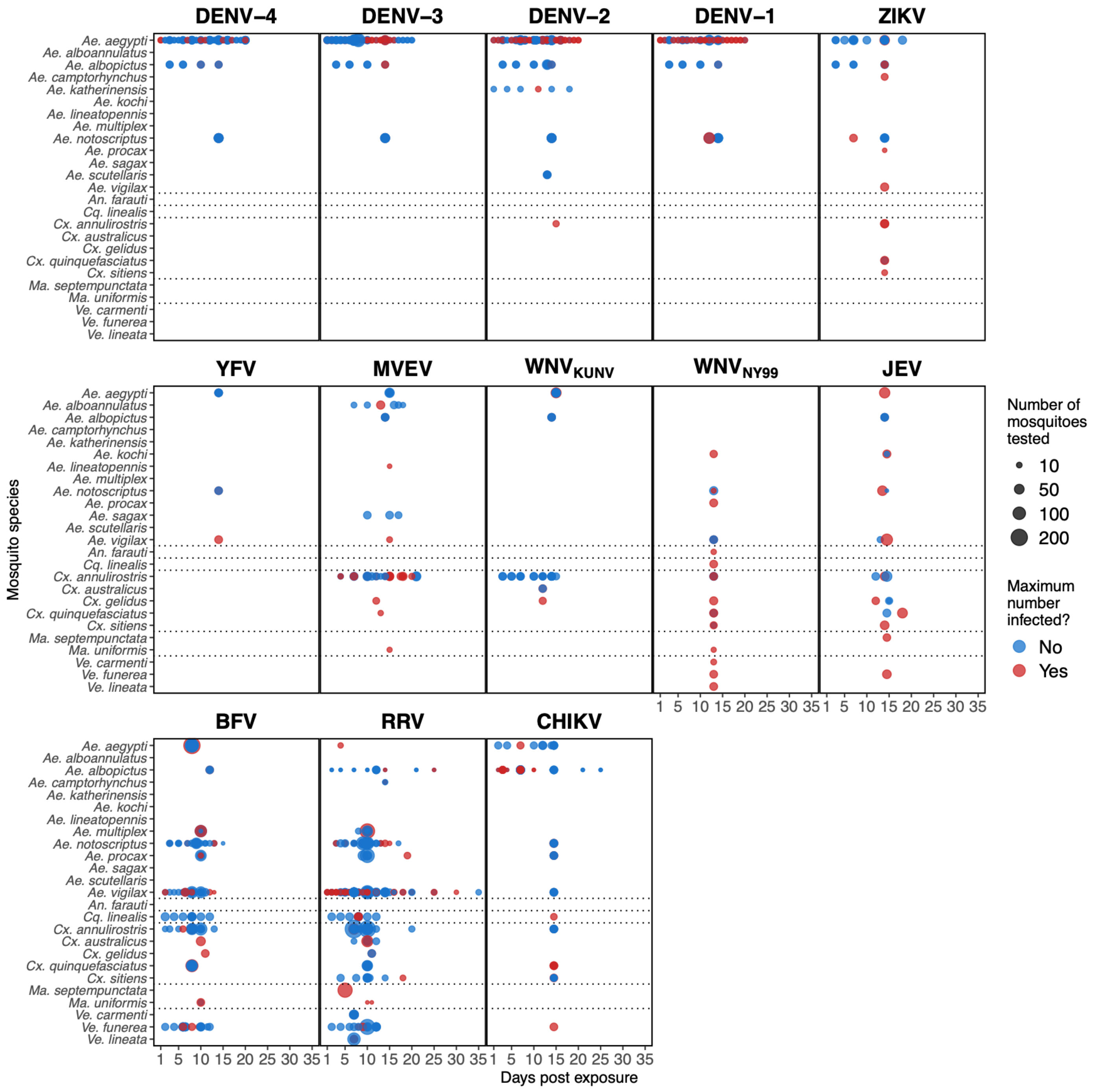

Figure S8. Days post viral exposure on which mosquitoes were tested for infection. Blue points show experimental days that did not lead to the highest detected proportion of infected mosquitoes; red points show the day[s] that resulted in the highest observed infected proportion.
