# Supplementary material for: Not all mosquitoes are created equal: A synthesis of vector competence experiments reinforces virus associations of Australian mosquitoes
Source: PLoS Negl Trop Dis. 2022 Oct 4;16(10):e0010768. doi: 10.1371/journal.pntd.0010768 (PMC9565724; doi:10.1371/journal.pntd.0010768)
Supplement: S10 Fig — Continuous functions for mosquito infection over dose (top), and for dissemination and transmission over time (middle and bottom, respectively). The results pictured here are from a generalized linear mixed effects model (GLMM) using Aedes aegypti for all viruses they have been tested with in the lab; however, here we only show the three viruses for which more than one data point exists for infection, dissemination, and transmission. For infection (top), medians (solid lines) and 95% confidence intervals (grey bands) are estimated using twelve days post exposure; for dissemination (middle) and transmission (bottom) estimates are drawn for a dose of 7 log10 IU/mL. (PDF) [file pntd.0010768.s010.pdf]

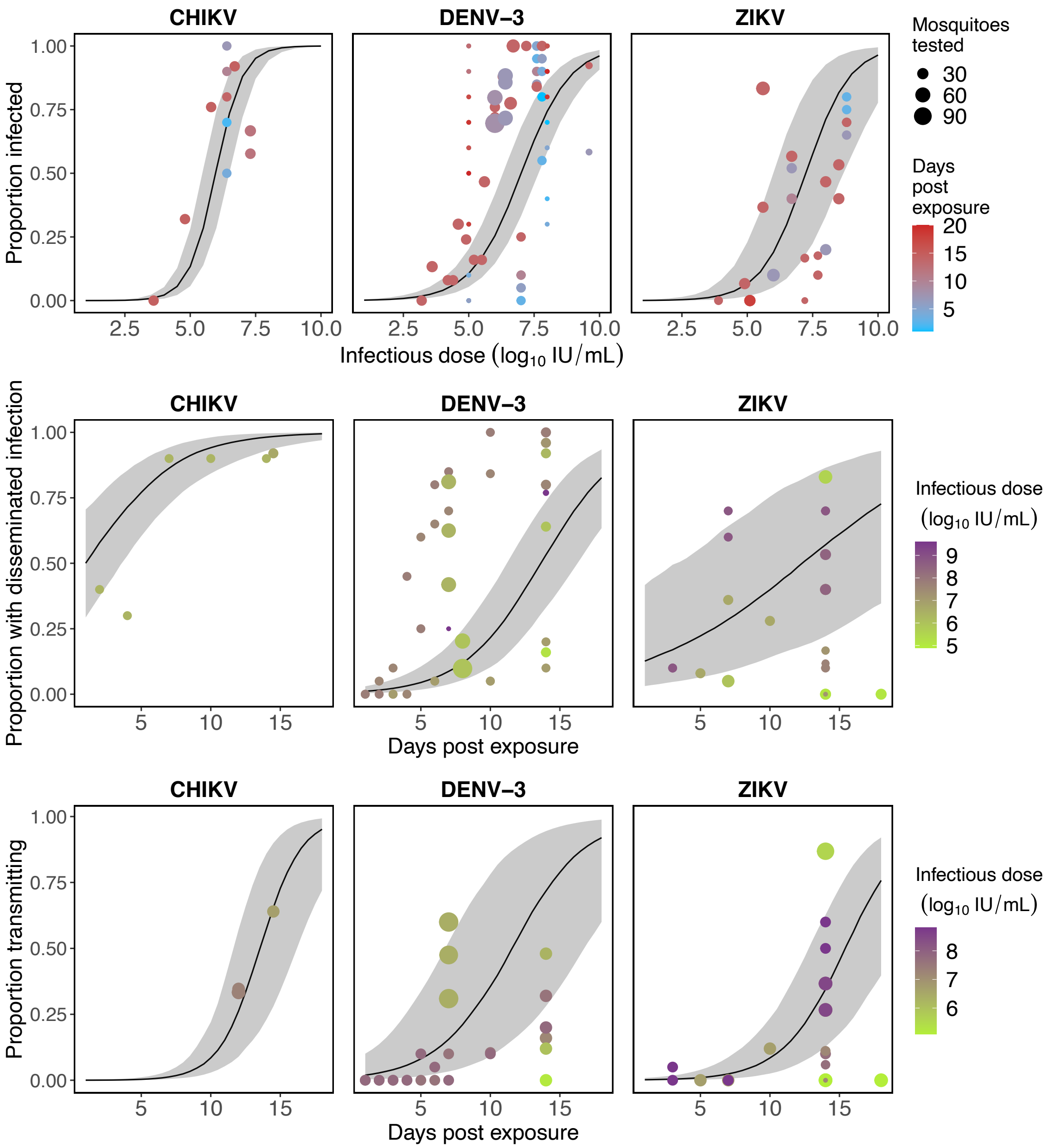

Figure S10. Continuous functions for mosquito infection over dose (top), and for dissemination and transmission over time (middle and bottom, respectively). The results pictured here are from a generalized linear mixed effects model (GLMM) using *Aedes aegypti* for all viruses they have been tested with in the lab; however, here we only show the three viruses for which more than one data point exists for infection, dissemination, and transmission. For infection (top), medians (solid lines) and 95% confidence intervals (grey bands) are estimated using twelve days post exposure; for dissemination (middle) and transmission (bottom) estimates are drawn for a dose of 7 $\log_{10}$ IU/mL.
