# Supplementary material for: Not all mosquitoes are created equal: A synthesis of vector competence experiments reinforces virus associations of Australian mosquitoes
Source: PLoS Negl Trop Dis. 2022 Oct 4;16(10):e0010768. doi: 10.1371/journal.pntd.0010768 (PMC9565724; doi:10.1371/journal.pntd.0010768)
Supplement: S11 Fig — Multiple points of the same color and shape within a viral group represent results from different viruses within that group. The dashed line is a 1:1 line; points beneath the line represent mosquito-virus pairings with a greater infection probability than transmission probability. (PDF) [file pntd.0010768.s011.pdf]

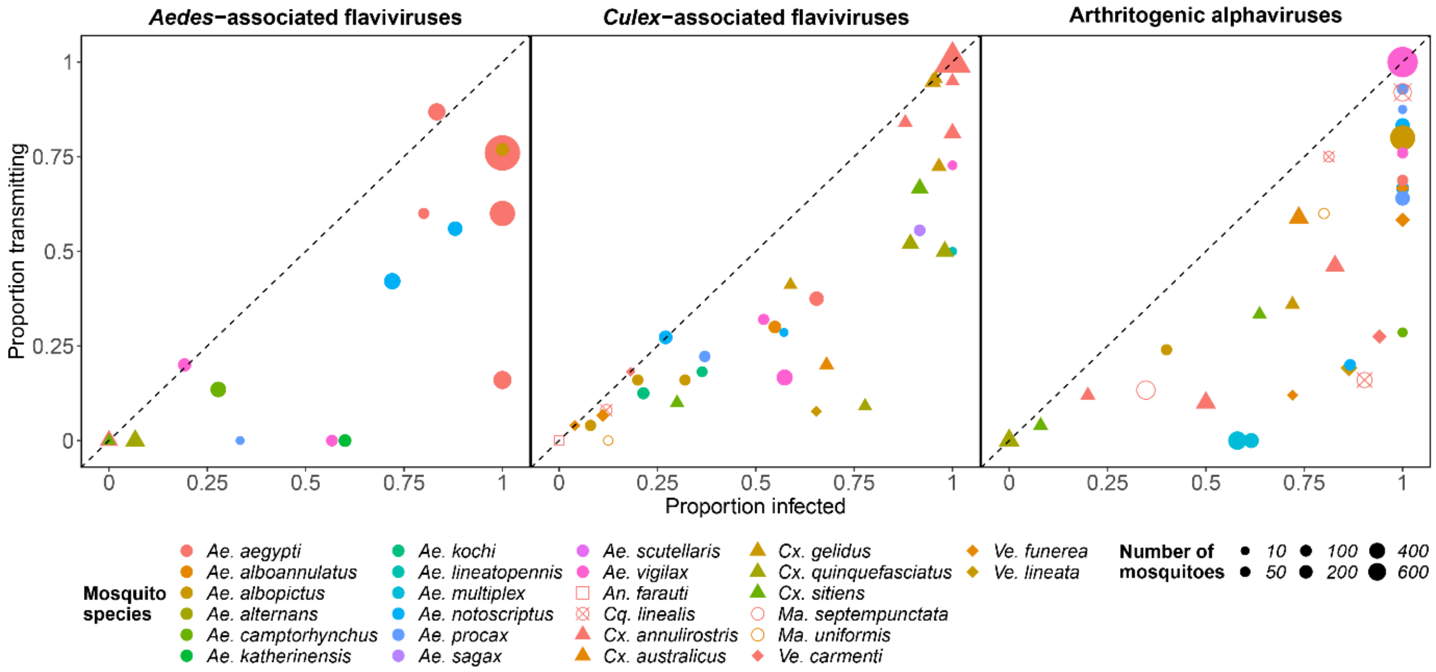

Figure S11. Infection and transmission probabilities for each mosquito-virus pairing tested. Multiple points of the same color and shape within a viral group represent results from different viruses within that group. The dashed line is a 1:1 line; points beneath the line represent mosquito-virus pairings with a greater infection probability than transmission probability.
