# Supplementary material for: Not all mosquitoes are created equal: A synthesis of vector competence experiments reinforces virus associations of Australian mosquitoes
Source: PLoS Negl Trop Dis. 2022 Oct 4;16(10):e0010768. doi: 10.1371/journal.pntd.0010768 (PMC9565724; doi:10.1371/journal.pntd.0010768)
Supplement: S12 Fig — Mosquito species frequently measured in infection experiments (total sample size across all experiments; X axis) were also more frequently measured in transmission experiments (total sample size across all experiments; Y axis). Each point for a mosquito species represents a different virus. (PDF) [file pntd.0010768.s012.pdf]

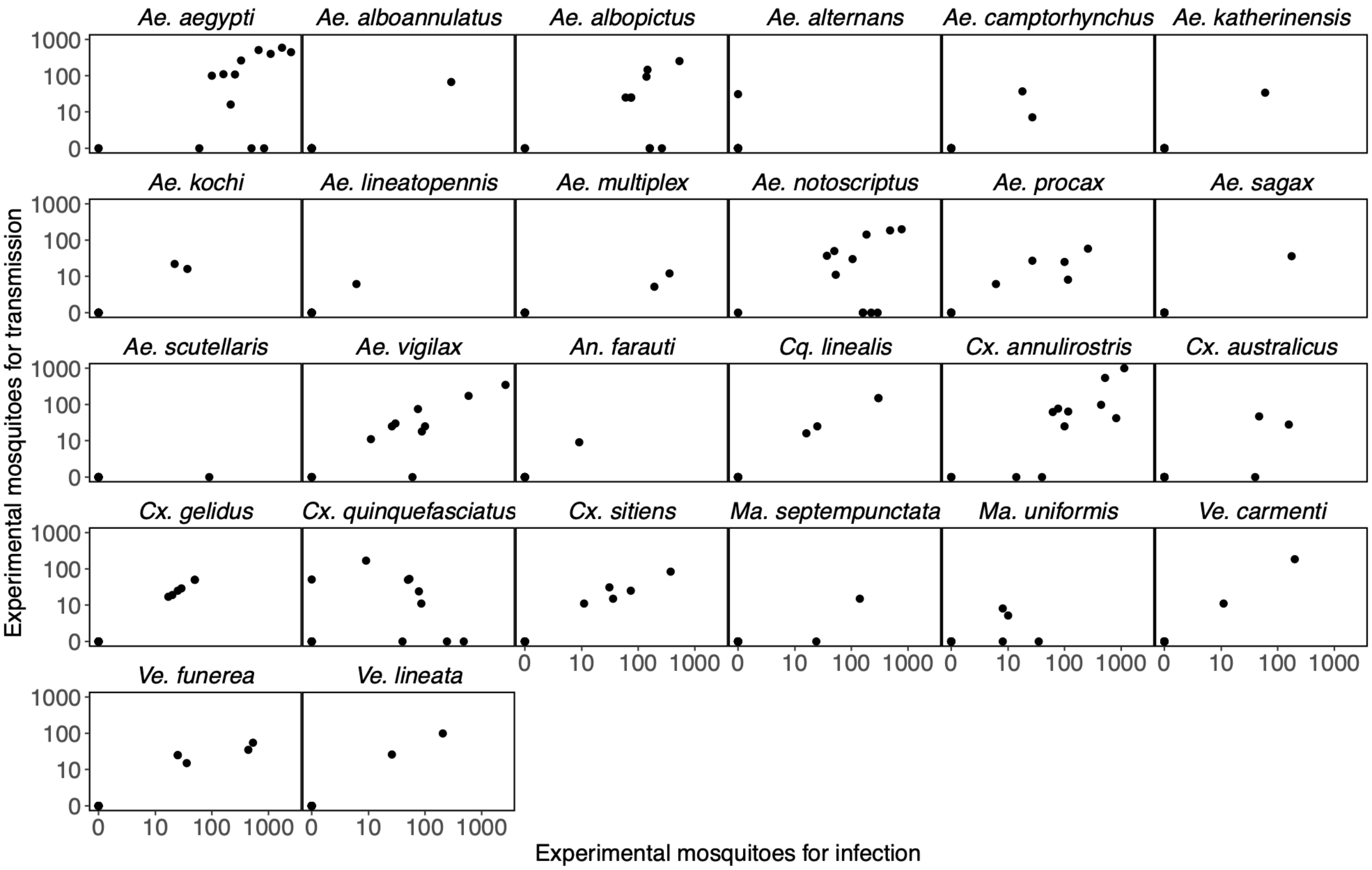

Figure S12. Mosquito species frequently measured in infection experiments (total sample size across all experiments; X axis) were also more frequently measured in transmission experiments (total sample size across all experiments; Y axis). Each point for a mosquito species represents a different virus.
