# Supplementary material for: Not all mosquitoes are created equal: A synthesis of vector competence experiments reinforces virus associations of Australian mosquitoes
Source: PLoS Negl Trop Dis. 2022 Oct 4;16(10):e0010768. doi: 10.1371/journal.pntd.0010768 (PMC9565724; doi:10.1371/journal.pntd.0010768)
Supplement: S13 Fig — Increased research effort led to a higher maximum proportion for infection, dissemination, and transmission (logistic regression, p < 0.05). Increased dose does not lead to a detectable increase in maximum proportion (logistic regression, p > 0.05). Each point represents a single virus-species pair. (PDF) [file pntd.0010768.s013.pdf]

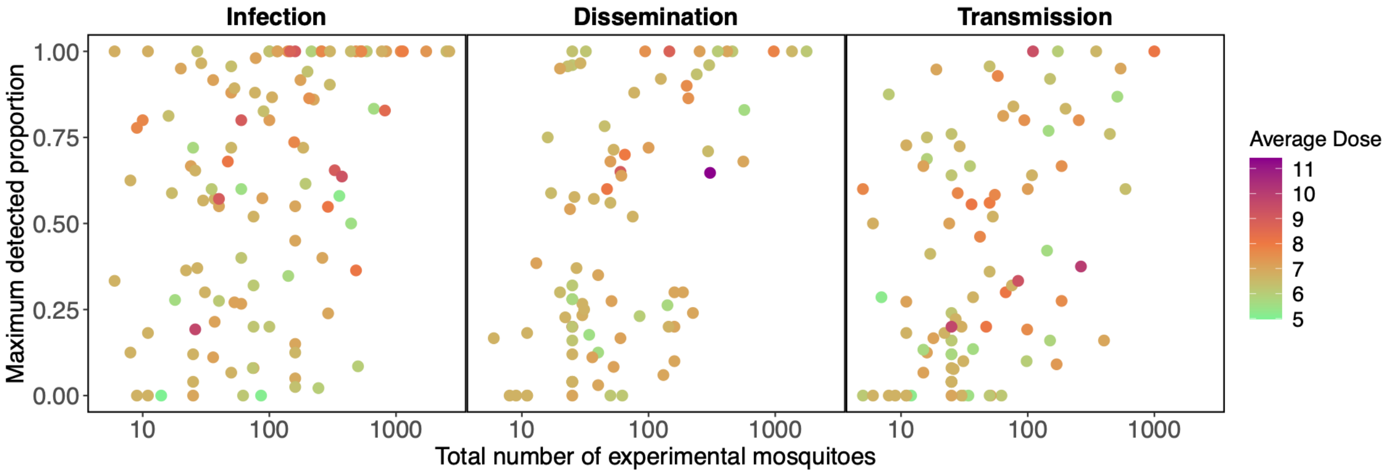

Figure S13. Increased research effort lead to a higher maximum proportion for infection, dissemination, and transmission (logistic regression, $p < 0.05$). Increased dose does not lead to a detectable increase in maximum proportion (logistic regression, $p > 0.05$). Each point represents a single virus-species pair.
