# Supplementary material for: Not all mosquitoes are created equal: A synthesis of vector competence experiments reinforces virus associations of Australian mosquitoes
Source: PLoS Negl Trop Dis. 2022 Oct 4;16(10):e0010768. doi: 10.1371/journal.pntd.0010768 (PMC9565724; doi:10.1371/journal.pntd.0010768)
Supplement: S1 Text — (PDF) [file pntd.0010768.s014.pdf]

Supplemental Results

Mosquito-virus associations using maxima; variation among virus group-mosquito genera

Aedes-*associated flaviviruses*

Aedes spp. varied strongly in their ability to become infected with different AAFVs, from a minimum of 3% of *Aedes notoscriptus* individuals becoming infected with DENV-4 to a maximum of 100% of *Ae. aegypti* individuals becoming infected with each DENV strain and 100% of *Aedes albopictus* individuals becoming infected with ZIKV (S3 Fig). Similar to the variation in infection, *Aedes* species also varied in their ability to transmit AAFVs, from zero percent of *Aedes katherinensis* transmitting DENV-2, or *Aedes procax* and *Aedes vigilax* transmitting ZIKV, to a maximum of 87% of *Ae. aegypti* transmitting ZIKV (S4 Fig). In contrast to the large variation among *Aedes* spp., none of the three *Culex* species (*Culex annulirostris*, *Culex quinquefasciatus*, and *Culex sitiens*) experimentally exposed to ZIKV were able to transmit the virus (S4 Fig) and only one individual *Cx. quinquefasciatus* (of 50 tested) was infected (S3 Fig). Similarly, *Cx. annulirostris* was refractory to infection with DENV-4 in the only experiment where Australian *Culex* spp. had been exposed to any of the DENVs (S3 Fig).

Culex-*associated flaviviruses*

Variation among the CAFVs was more difficult to generalize than that of the other viral groups. MVEV had the highest average overall infection and transmission proportions (78% and 53%, respectively) of all viruses within the group, and one of the highest across all viruses (S3 Fig, S4 Fig). By contrast, WNV (New York 1999 strain; WNV_{NY99}) had the lowest infection and transmission proportions (43% and 26%, respectively) of all CAFVs (S3 Fig, S4 Fig). *Cx. annuliros-*

tris and *Culex gelidus* transmitted the CAFVs at rates above 40% (S4 Fig). Although not as susceptible as *Culex* species for the CAFVs, the majority of *Aedes* spp. including *Ae. aegypti*, *Ae. vigilax*, *Aedes sagax,* and *Aedes lineatopennis* demonstrated relatively high infection proportions (>50%) with MVEV (S3 Fig). All examined *Verrallina* and *Mansonia* species were susceptible to infection with CAFVs, but in almost all cases, the proportion infected and transmitting these viruses were lower than *Culex* spp. (S3 Fig, S4 Fig).

Arthritogenic alphaviruses

The high susceptibility to infection and subsequent transmission of arthritogenic alphaviruses by most Australian mosquitoes (S3 Fig, S4 Fig) potentially points to a general virus genus attribute, especially given the high number of mosquito species (19 out of 27) representing different genera that were exposed to these viruses. *Aedes* spp. were generally highly susceptible to infection and readily transmitted most arthritogenic arboviruses (S3 Fig, S4 Fig). For example, both *Ae. aegypti* and *Ae. albopictus* were highly susceptible to RRV and CHIKV, and readily transmitted these viruses at rates above 60%. However, both species had low infection proportions for BFV (< 9% and ≤ 40%, respectively; S3 Fig). There was more variation among *Culex* spp. in competence for arthritogenic alphaviruses than for other mosquito genera. All species of *Culex* were highly susceptible to infection with RRV (> 60%) but had very low susceptibility to CHIKV and BFV (S3 Fig). All *Culex* spp. were capable of transmitting RRV, although they had a much lower probability than either *Aedes* or *Coquillettidia*. *Verrallina* were highly susceptible to infection with all anthritogenic alphaviruses, but like *Culex*, had lower transmission proportions than other mosquito genera tested (S3, S4 Fig).
