# Supplementary material for: Not all mosquitoes are created equal: A synthesis of vector competence experiments reinforces virus associations of Australian mosquitoes
Source: PLoS Negl Trop Dis. 2022 Oct 4;16(10):e0010768. doi: 10.1371/journal.pntd.0010768 (PMC9565724; doi:10.1371/journal.pntd.0010768)
Supplement: S3 Fig — A) Maximum proportion of mosquitoes positive for infection across all mosquito species tested for each virus (median and 80% quantiles across all mosquitoes). B) Maximum proportion of each mosquito species found positive for infection among all of the laboratory experiments for each mosquito-virus pair (represented by color and the bold-face top number in each box). The number of experimental mosquitoes tested in the study[s] in which the maximum proportion was observed is displayed in square brackets (in the case of multiple experiments finding the same maximum, the sample sizes were summed). Viruses are organized into three groups: Aedes-associated flaviviruses (yellow fever [YFV], Zika [ZIKV] and dengue [DENV, separated by serotype]); Culex-associated flaviviruses (Japanese encephalitis [JEV], West Nile New York strain [WNVNY99], West Nile Kunjin strain [WNVKUNV] and Murray Valley encephalitis [MVEV]); and arthritogenic alphaviruses (chikungunya [CHIKV], Ross River [RRV] and Barmah Forest [BFV]). Vertical dotted lines separate mosquito genera, from left to right: Aedes [Ae], Anopheles [An], Coquillettidia [Cq], Culex [Cx], Mansonia [Ma] and Verrallina [Ve]. (PDF) [file pntd.0010768.s003.pdf]

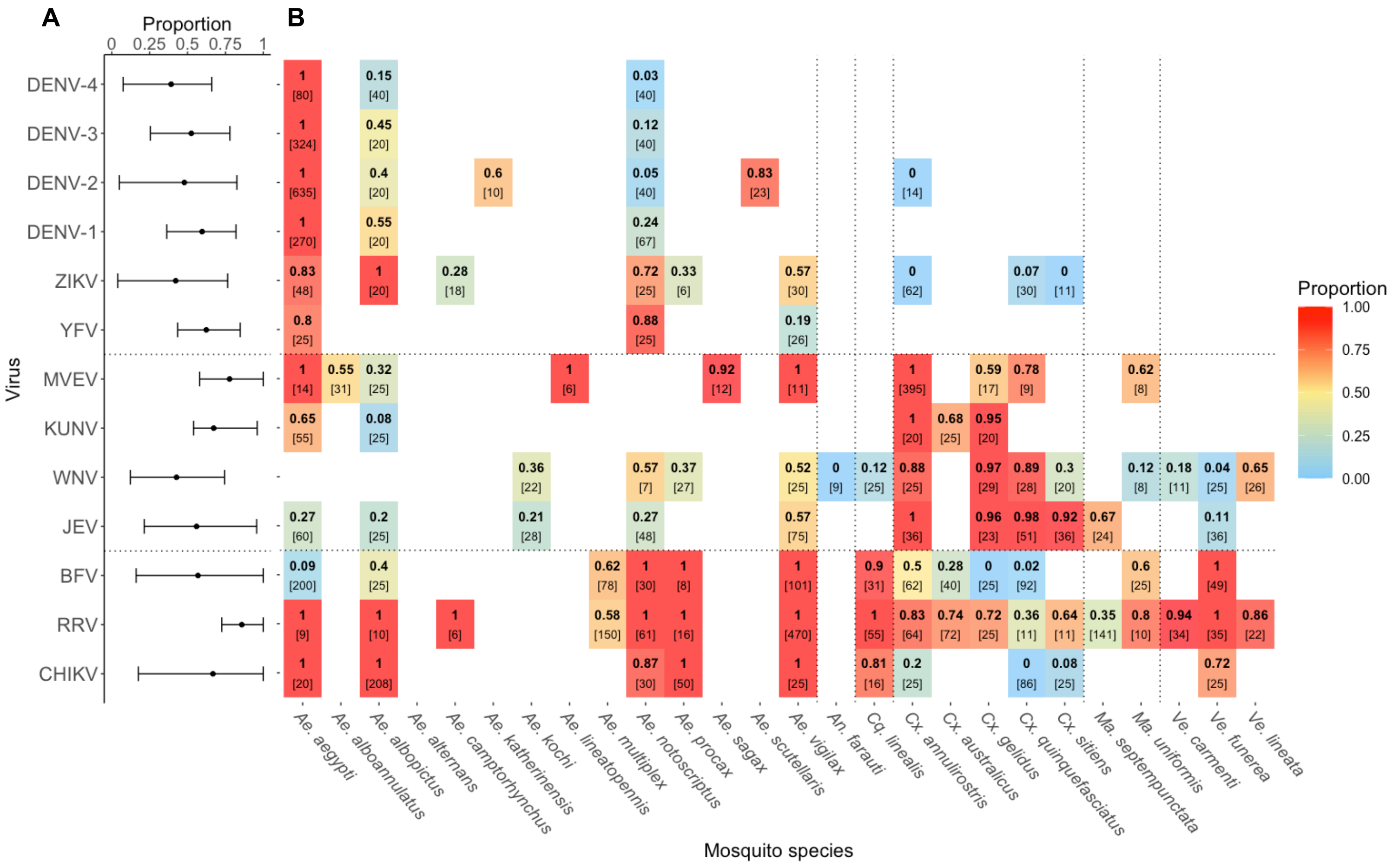

Figure S3. A) Maximum proportion of mosquitoes positive for infection across all mosquito species tested for each virus (median and 80% quantiles across all mosquitoes). **B)** Maximum proportion of each mosquito species found positive for infection among all of the laboratory experiments for each mosquito-virus pair (represented by color and the bold-face top number in each box). The number of experimental mosquitoes tested in the study[s] in which the maximum proportion was observed is displayed in square brackets (in the case of multiple experiments finding the same maximum, the sample sizes were summed). Viruses are organized into three groups: *Aedes*-associated flaviviruses (yellow fever [YFV], Zika [ZIKV] and dengue [DENV, separated by serotype]); *Culex*-associated flaviviruses (Japanese Encephalitis [JEV], West Nile New York strain [WNV_{NY99}], West Nile Kunjin strain [WNV_{KUNV}] and Murray Valley encephalitis [MVEV]); and arthritogenic alphaviruses (chikungunya [CHIKV], Ross River [RRV] and Barmah Forest [BFV]). Vertical dotted lines separate mosquito genera, from left to right: *Aedes* [*Ae*], *Anopheles* [*An*], *Coquillettidia* [*Cq*], *Culex* [*Cx*], *Mansonia* [*Ma*] and *Verrallina* [*Ve*].
