# Supplementary material for: Not all mosquitoes are created equal: A synthesis of vector competence experiments reinforces virus associations of Australian mosquitoes
Source: PLoS Negl Trop Dis. 2022 Oct 4;16(10):e0010768. doi: 10.1371/journal.pntd.0010768 (PMC9565724; doi:10.1371/journal.pntd.0010768)
Supplement: S4 Fig — A) Maximum proportion of mosquitoes positive for transmission across all mosquito species tested for each virus (median and 80% quantiles across all mosquitoes). B) Maximum proportion of each mosquito species found positive for transmission among all of the laboratory experiments for each mosquito-virus pair (represented by color and the bold-face top number in each box). The number of experimental mosquitoes tested in the study[s] in which the maximum proportion was observed is displayed in square brackets (in the case of multiple experiments finding the same maximum, the sample sizes were summed). Viruses are organized into three groups and mosquito species are grouped into genera as in Fig 2. (PDF) [file pntd.0010768.s004.pdf]

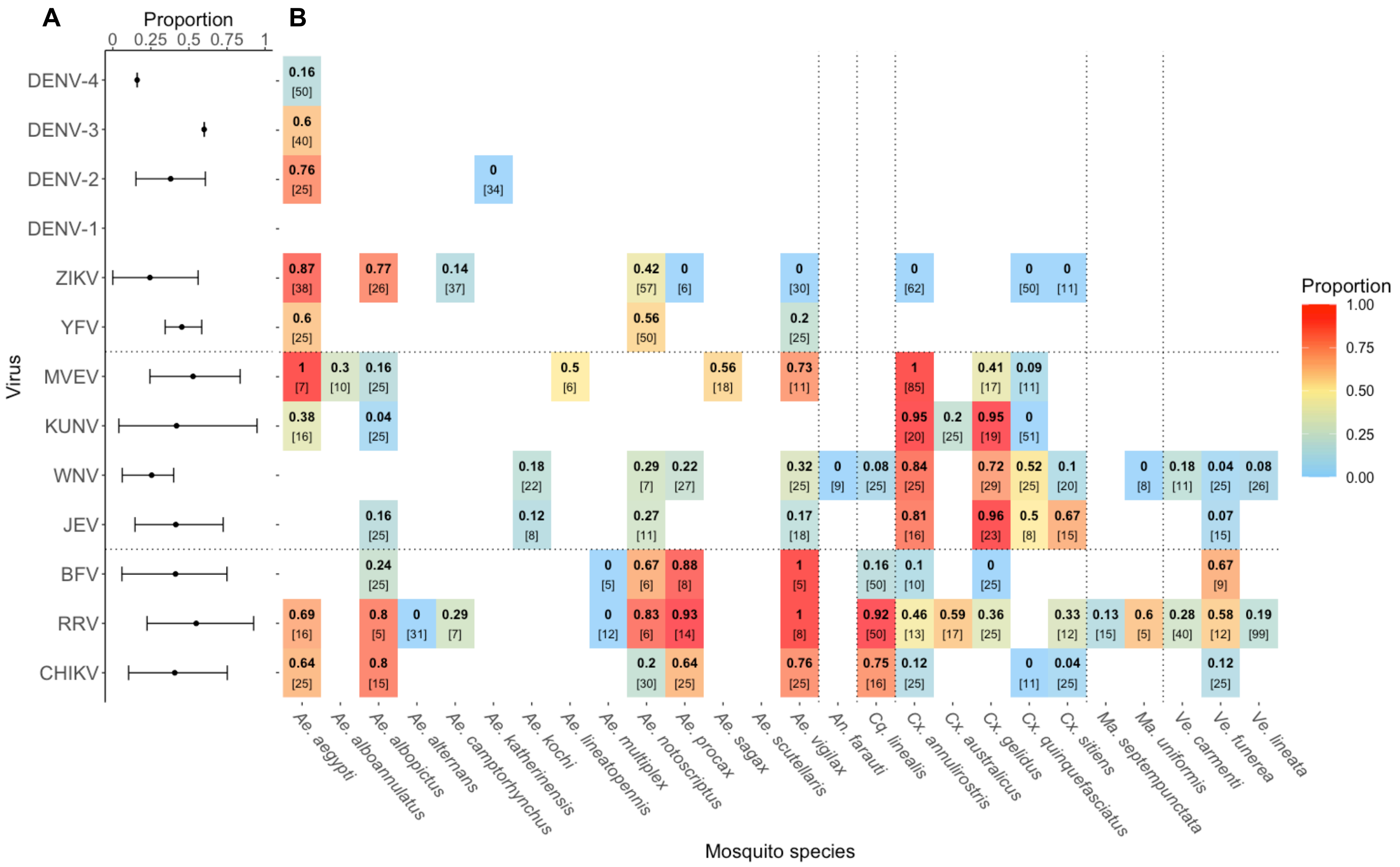

Figure S4. A) Maximum proportion of mosquitoes positive for transmission across all mosquito species tested for each virus (median and 80% quantiles across all mosquitoes). **B)** Maximum proportion of each mosquito species found positive for transmission among all of the laboratory experiments for each mosquito-virus pair (represented by color and the bold-face top number in each box). The number of experimental mosquitoes tested in the study[s] in which the maximum proportion was observed is displayed in square brackets (in the case of multiple experiments finding the same maximum, the sample sizes were summed). Viruses are organized into three groups and mosquito species are grouped into genera as in Figure 2.
